# A Porcine Model of Heart Failure With Preserved Ejection Fraction Induced by Chronic Pressure Overload Characterized by Cardiac Fibrosis and Remodeling

**DOI:** 10.3389/fcvm.2021.677727

**Published:** 2021-06-02

**Authors:** Weijiang Tan, Xiang Li, Shuang Zheng, Xiaohui Li, Xiaoshen Zhang, W. Glen Pyle, Honghua Chen, Jian Wu, Huan Sun, Yunzeng Zou, Peter H. Backx, Feng Hua Yang

**Affiliations:** ^1^Guangdong Provincial Key Laboratory of Laboratory Animals, Guangdong Laboratory Animals Monitoring Institute, Guangzhou, China; ^2^Department of Cardiovascular Surgery, The First Affiliated Hospital, Jinan University, Guangzhou, China; ^3^Department of Biomedical Sciences, University of Guelph, Guelph, ON, Canada; ^4^Shanghai Institute of Cardiovascular Diseases, Zhongshan Hospital and Institutes of Biomedical Sciences, Fudan University, Shanghai, China; ^5^Cardiology Department, China-Japan Union Hospital of Jilin University, Changchun, China; ^6^Department of Biology, York University, Toronto, ON, Canada; ^7^Department of Physiology, University of Toronto, Toronto, ON, Canada

**Keywords:** porcine model, pressure overload, heart failure with preserved ejection fraction, fibrosis, signal transduction, cardiac remodeling

## Abstract

Heart failure is induced by multiple pathological mechanisms, and current therapies are ineffective against heart failure with preserved ejection fraction (HFpEF). As there are limited animal models of HFpEF, its underlying mechanisms have not yet been elucidated. Here, we employed the descending aortic constriction (DAC) technique to induce chronic pressure overload in the left ventricles of Tibetan minipigs for 12 weeks. Cardiac function, pathological and cellular changes, fibrotic signaling activation, and gene expression profiles were explored. The left ventricles developed concentric hypertrophy from weeks 4 to 6 and transition to dilation starting in week 10. Notably, the left ventricular ejection fraction was maintained at >50% in the DAC group during the 12-week period. Pathological examination, biochemical analyses, and gene profile analysis revealed evidence of inflammation, fibrosis, cell death, and myofilament dephosphorylation in the myocardium of HFpEF model animals, together with gene expression shifts promoting cardiac remodeling and downregulating metabolic pathways. Furthermore, we noted the activation of several signaling proteins that impact cardiac fibrosis and remodeling, including transforming growth factor-β/SMAD family members 2/3, type I/III/V collagens, phosphatidylinositol 3-kinase, extracellular signal-regulated kinase, matrix metalloproteinases 2 and 9, tissue inhibitor of metalloproteinases 1 and 2, interleukins 6 and 1β, and inhibitor of κBα/nuclear factor-κB. Our findings demonstrate that this chronic pressure overload-induced porcine HFpEF model is a powerful tool to elucidate the mechanisms of this disease and translate preclinical findings.

## Introduction

Although survival and hospitalization rates for patients with heart failure (HF) have greatly improved over the past three decades, HF remains one of the leading causes of death worldwide. European data reported that the 12-month all-cause mortality rate for hospitalized patients with HF is approximately 24% (1). In the USA, an estimated 6.2 million adults 20 years of age or older had HF between 2013 and 2016 (2). In China, among the 290 million patients with cardiovascular disease, approximately 2.5 million suffer from HF (3). The European Society of Cardiology (ESC) guidelines on the diagnosis and treatment of acute and chronic HF classify the disease as either heart failure with reduced ejection fraction [HFrEF, with a left ventricular ejection fraction (LVEF) ≤40%], mid-range EF (HFmrEF, LVEF 40–49%), or preserved EF (HFpEF, LVEF ≥50%) (4). The USA and Japan have adopted similar guidelines (5, 6). Based on studies of multiple diverse community-based cohorts in the USA, HFpEF accounts for 51–63% of HF cases (7); however, the prevalence is lower in Asian countries, at ~30% (8). Clinically, the criteria for HFpEF diagnosis in the 2016 ESC guidelines include symptoms and signs, LVEF ≥50%, elevated natriuretic peptides [brain natriuretic peptide (BNP) and N terminal proBNP], and relevant structural heart disease and/or diastolic dysfunction (4). Additionally, patients with HFpEF display cardiac vasculature abnormalities, inflammation, fibrosis, ischemia, metabolic disorder, myocyte hypertrophy, and detrimental cellular signal transduction (7, 9, 10). However, because of limitations in available animal models, these mechanisms remain hypothetical.

To explore safe and more effective strategies to treat and manage HF, numerous approaches have been used to generate animal models that mimic its phenotypes (11, 12). Aortic constriction-induced HF in rodents has been one of the most widely used models since its development in the early 1990s (13–15). Aortic constriction mimics high blood pressure, inducing chronic pressure overload in the left ventricles and ventricular hypertrophy or dilation (13, 16). Experimental pressure overload can induce all three types of HF. Recently, researchers successfully developed a HFpEF model by controlling the degree of vessel narrowing or blood flow. By using a constriction ring of a specific size (inner diameter: 0.71 mm), they could induce cardiac hypertrophy with preserved EF (>50%) in mice for 8–20 weeks (17). In pigs, an HF model with cardiac hypertrophy and preserved EF can be generated by placing an inflatable cuff at the aortic root to increase left ventricle pressure (18). However, compared with mice, the phenotypes and mechanisms of pig HFpEF models remain relatively unreported.

In this study, we employed the aortic constriction technique to induce chronic pressure overload in porcine ventricles. The pigs developed left ventricular concentric hypertrophy, followed by left ventricle dilation with a preserved ejection fraction. Cardiac remodeling, histology, gene expression profiles, and fibrotic signaling were explored. This is the first study to use descending aortic constriction (DAC) to model HFpEF, providing a powerful tool to study this condition.

## Methods

### Ethical Statement and Animals

All animal experiments were performed in accordance with the Guide for the Care and Use of Laboratory Animals (8th Ed., 2011, The National Academies, USA). The protocol was approved by the Institutional Animal Care and Use Committee of the Guangdong Laboratory Animals Monitoring Institute (approval no. IACUC2017009). Ten Tibetan minipigs (male, 25–30 kg) were purchased from a licensed laboratory animal facility [license no. SCXK (YUE) 2015-0036, China]. Minipigs were randomly assigned to two groups (*n* = 5 each). One group underwent sham surgery; the second underwent DAC surgery. During the experimental period, the animals were housed in an AAALAC-accredited facility at the Guangdong Laboratory Animals Monitoring Institute [license no. SYXK (YUE) 2016-0122, China]. At this facility, the ambient temperature and humidity were 20–26°C and 40–70%, respectively, and the light cycle was 12 h day/12 h night.

Before (week 0) and after surgery (weeks 2, 4, 6, 8, 10, and 12), echocardiography was performed to evaluate cardiac morphology and function. Twelve weeks after surgery, animals were euthanized, and cardiac tissues were collected for histopathological and expression analyses.

### DAC Model

#### Surgical Procedure

The descending aorta segment was selected as the constriction site. All surgical instruments were autoclaved, and aseptic techniques were applied during the surgical procedure. The operating room was located in the AAALAC-accredited facility of the Guangdong Laboratory Animals Monitoring Institute. The minipigs were fasted overnight before surgery. Ketamine hydrochloride (6 mg/kg) and midazolam (0.5 mg/kg) were used for basal anesthesia, propofol (5 mg/kg) was used for induction anesthesia, and volatile anesthetic with isoflurane (1.5–2.5%) was used to maintain general anesthesia. The animals were placed in the right lateral position and connected to a veterinary monitor (iPM12Vet; Mindray, China). After pre-operative skin preparation, a ~15 cm-long incision was made on the thoracic skin. Electrosurgical and blunt dissection techniques were used in combination to separate the muscle layers, and the constriction site (the descending segment of the aorta, located under the fourth to fifth intercostal space) was exposed.

#### Pressure Determination at the Constriction Site

**Figure 1:**
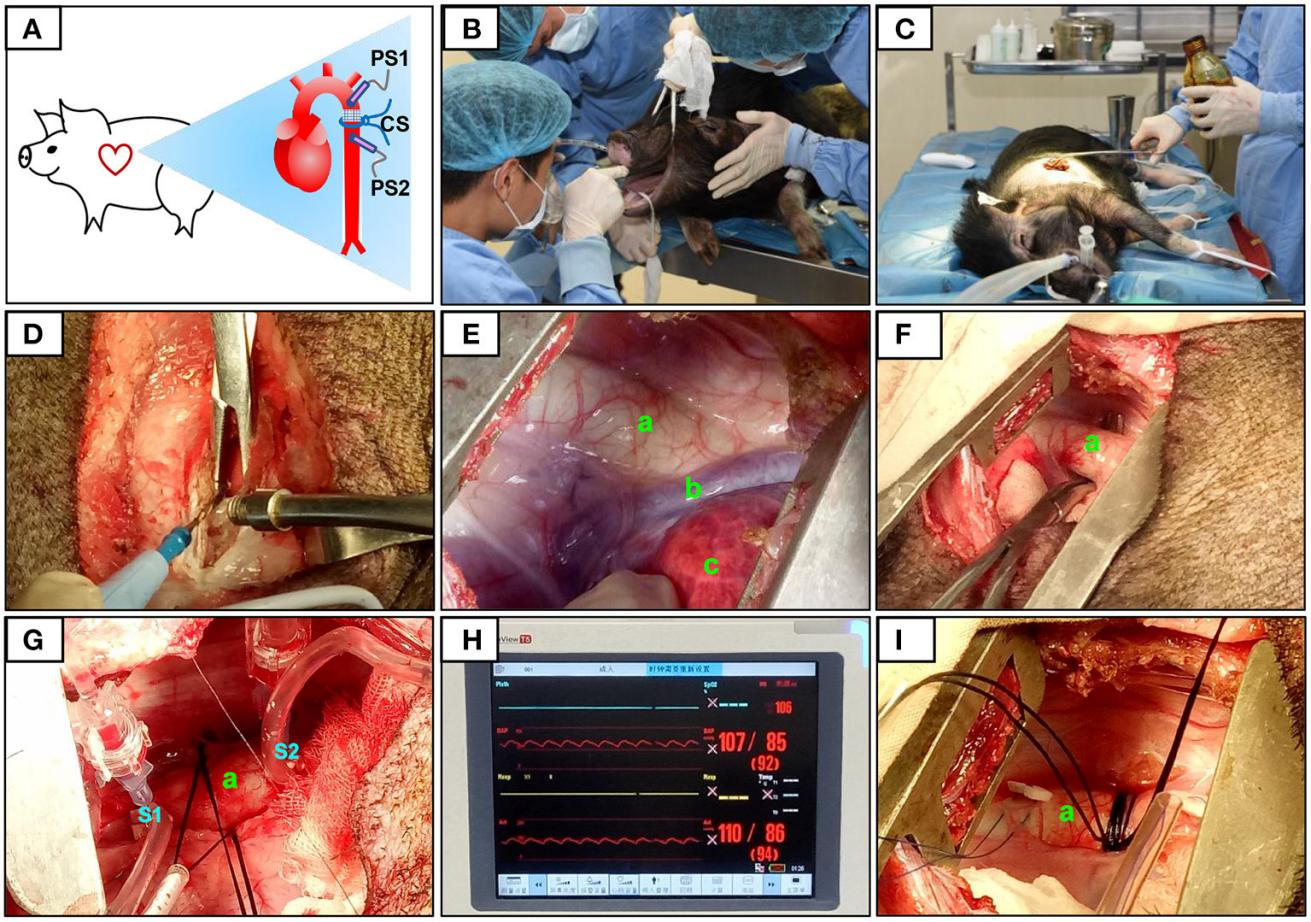
Surgical procedure and pressure determination at the constriction site. The constriction site (CS) was at the descending aorta segment **(A)**. During the surgery, volatile anesthetic with isoflurane was used for the maintenance of general anesthesia **(B)**. After pre-operative skin preparation **(C)**, an incision was made and a combination of electrosurgical and blunt dissection techniques was used to separate muscle layers **(D)**. The descending segment of the aorta located under the fourth to fifth intercostal space was exposed **(E,F)**. After determining the constriction site, two pressure sensors were placed on the segment proximal to the heart (S1) and after (on the segment distal to the heart, S2) the constriction site **(G)**. The pressures at S1 and S2 were displayed over the ECG monitor **(H)**. When the pressure at S1 (PS1) reached 120% of that at S2 (PS2), the sutures were then secured with surgical knots **(I)**. The aorta (a), accessory hemiazygos vein (b), and lung (c) were shown in **(E–G,I)**.

After determining the constriction site, two pressure sensors were placed on the segment proximal to the heart (S1) and after the constriction site (on the segment distal to the heart, S2; Figure 1). The pressures at S1 and S2 were equal and designated P0. At the constriction site, three layers of sterile gauze were wrapped around the aorta, and surgical sutures (size 7–0) were placed. Pressure was monitored as the sutures were gradually tightened. When the pressure at S1 (PS1) reached 120% of that at S2 (PS2), this condition was stabilized for 30 s, and the sutures were secured with surgical knots. After inserting an indwelling chest tube, the muscles and skin were closed layer by layer. Penicillin was applied to the operation site post-surgery and daily for 1 week afterwards (20,000 U/kg). Animals in the sham group also underwent thoracotomy, but no aortic constriction was performed.

### Echocardiography

An ultrasound diagnostic imaging system equipped with a Cardiac PA122 phased array transducer (3–8 MHz, MyLab30; Esatoe, Italy) was used to evaluate cardiac morphology and function at weeks 0, 2, 4, 6, 8, 10, and 12. Following anesthesia with ketamine hydrochloride (50 mg/kg), a transducer was placed in the left or right intercostal area to obtain both B-mode and M-mode images of the left ventricular long axis, and internal aortic dimensions. The measured indices of the M-mode images included the left ventricular dimensions at the end of systole and diastole (LVIDs and LVIDd, respectively), the ventricular septum thickness at the end of systole and diastole (VSTs and VSTd, respectively), the posterior wall thickness at the end of systole and diastole (PWTs and PWTd, respectively), and the aortic root diameter at the end of systole and diastole (ARDs and ARDd, respectively). The left ventricular EF and fraction shortening (FS) were analyzed using software provided by Esaote Inc.

### Serum Biochemistry

After overnight fasting, blood samples (5 mL) were collected from the minipigs and allowed to clot for 60 min at room temperature. Serum was separated by centrifugation at 2,150 × *g* at 4°C for 30 min. For serum biochemistry tests, an automatic biochemistry analyzer (LX20; Beckman, USA) and corresponding reagents were used. Laboratory parameters measured included creatine kinase (CK), lactate dehydrogenase (LDH), and creatinine.

### Hematoxylin and Eosin (HE) and Sirius Red Staining

HE staining was used to examine tissue damage to the main organs, and Sirius red staining was used to determine cardiac fibrosis. Animals were euthanized with ketamine hydrochloride at the end of the experimental period. Heart tissues (left ventricle, right ventricle, left atrium, right atrium, and interventricular septum), aortic vessels, and the lungs, liver, and kidneys were collected. Tissue blocks were fixed overnight in 4% paraformaldehyde. The tissues were embedded in paraffin and sectioned to 3 μm, then sequentially treated with xylene, absolute ethanol, 95% absolute ethanol, 80% absolute ethanol, 70% absolute ethanol, and pure water for dewaxing. For HE staining, the sections were incubated in hematoxylin solution (cat. no. H3136; Sigma-Aldrich, USA) for 10 min and then with alcohol-soluble eosin (cat. no. E4009; Sigma-Aldrich, USA) for 25 s. For Sirius red staining to detect the myocardial collagen content, left ventricle sections were incubated in Sirius red solution for 1 h and then washed with acidified water. Following dehydration and mounting, the tissue sections were observed under a microscope (DM2500; Leica, Germany).

### Enzyme-Linked Immunosorbent Assay (ELISA)

A commercial ELISA kit (Cusabio Biotech Co., China) was used to determine serum concentrations of cardiac troponin I (cTnI) at week 12. Blood samples from the minipigs were centrifuged at 2,500 × *g* at 4°C for 30 min, and the supernatants were collected. The ELISA was performed according to the manufacturer's instructions. Optical density was measured at 450 nm using a microplate reader (Multiskan FC; Thermo Fisher Scientific, USA).

### RNA Sequencing, Raw Data Processing, and Gene Set Enrichment Analysis (GSEA)

RNA sequencing was used to profile gene expression following DAC treatment as previously described (19). Briefly, total RNA was extracted using TRIzol reagent (cat. no.15596026; Invitrogen, USA). RNA concentrations were determined using an Invitrogen Qubit RNA Assay Kit on a Qubit 2.0 Fluorometer (cat. no. Q10210; Thermo Fisher Scientific, USA), and RNA integrity was tested using an RNA Nano 6000 Assay Kit (cat. no. 5067-1511; Agilent, USA) with the Bioanalyzer 2100 system (Agilent Technologies, USA). For RNA library generation, 3 μg of RNA per sample was used as input material After the addition of index codes and sample cluster generation, the libraries were sequenced on an Illumina HiSeq platform, and 125-bp/150-bp paired-end reads were generated. Gene reads from the sham and DAC groups were compared, and fold changes in expressed genes were log2-transformed. Differentially expressed genes (DEGs) were identified by *P*-values < 0.05. Furthermore, DEGs with log2 (fold change) values >0 and <0 were considered upregulated and downregulated, respectively. Gene expression profiles are presented using volcano plots. Up- and downregulated DEGs were inputted into the web-accessible gene annotation and analysis tool Metascape (https://metascape.org) to analyze enrichment in biological processes, and Database for Annotation, Visualization and Integrated Discovery (DAVID) v6.8 (https://david.ncifcrf.gov) to analyze Kyoto Encyclopedia of Genes and Genomes (KEGG) pathways in chronic pressure-overloaded hearts. Raw RNA-seq data were deposited in the National Center for Biotechnology Information Gene Expression Omnibus (https://www.ncbi.nlm.nih.gov/geo/) with accession number GSE167643.

### Terminal Deoxynucleotidyl Transferase (TdT) dUTP Nick-End Labeling (TUNEL) Staining

To examine myocardial apoptosis, left ventricle tissues were embedded in paraffin wax. After dewaxing and sectioning, the slices were incubated with proteinase K (20 μg/mL) at 37°C for 30 min. Sections were then washed with phosphate-buffered saline (pH 7.4) and treated with 0.5% Triton X-100 at room temperature for 5 min. A TUNEL cell apoptosis detection kit containing TdT (cat. no. Bio-C1088; Biyuntian, China) was used to fully cover the tissue, and samples were incubated in the dark for 60 min. The sections were then mounted with a 4′, 6-diamidino-2-phenylindole reagent containing an antifluorescence quencher (cat. no. P36966; Thermo Fisher Scientific, USA) and observed under a microscope (DM2500; Leica, Germany).

### Reverse Transcription Quantitative Polymerase Chain Reaction (RT-qPCR)

RT-qPCR was used to determine the expression of atrial natriuretic peptide (ANP), BNP, α-smooth muscle actin (SMA), tissue inhibitor of metalloproteinase 2 (TIMP2), and matrix metalloproteinase 9 (MMP9), which are associated with HF or fibrosis. Briefly, total RNA extracted from left ventricle tissue was subjected to RT-qPCR using the primers listed in Table 1. TB Green Premix Ex Taq II (cat. no. RR820; Takara Bio Inc., Japan) was used for qPCR as follows: 95°C for 30 s, then 40 cycles of 95°C for 5 s and 60°C for 34 s, then a final extension at 72°C for 10 min. Transcript levels were normalized to the expression of β-actin, and the results are expressed as ratios of the DAC group to the sham group.

**Table 1:** RT-qPCR Primers used in this study.

**Gene**	**Forward**	**Reverse**
MMP9	AGCCGCTCAGCAAGAAGATT	GCCCAGGCCCAACTTATCC
TIMP2	TTTTGCAATGCAGACGTAGTGA	GGGTTGCCGTAGATGTCGTT
α-SMA	CTGCCCTGGTGTGTGACAAT	TCCCACAATGGACGGGAAAA
ANP	GACCACTTGGAGGACAAGATG	TCCTCATTCTGCTCGCTTAGT
BNP	CTCAGAACTGCCAGGGATAC	GAGGACTTGGAAGATGCTACTG
β-actin	GACGATATTGCTGCGCTCGTG	TCAGGATGCCTCTCTTGCTCT

### Western Blotting

The cytosolic fraction was extracted from cardiac tissues of the sham and DAC groups to detect the expression levels of proteins involved in fibrosis, cellular proliferation, and inflammatory responses. Briefly, cytosolic proteins (40 μg) were resolved by 10% sodium dodecyl sulfate-polyacrylamide gel electrophoresis (SDS-PAGE) and transferred to polyvinylidene difluoride membranes (Millipore, USA). After blocking with 5% skim milk, the membranes were incubated with primary antibodies overnight at 4°C, followed by incubation with a species-appropriate secondary antibody. All primary and secondary antibodies were purchased from Cell Signaling Technology (Danvers, USA). The bands were detected with Immobilon Western Chemiluminescent HRP Substrate (Millipore, USA). Band density was analyzed using ImageJ (National Institutes of Health, Bethesda, MD, USA). Primary antibodies used were as follows: anti-collagen α1 (COL1α1; cat. no. 91144), anti-phospho-SMAD family member (SMAD)2 (p-SMAD2; cat. no. 18338T), anti-phospho-SMAD2/SMAD3 (p-SMAD2/3; cat. no. 8828S), anti-transforming growth factor β (TGF-β; cat. no. 3709S), anti-phospho-nuclear factor-κB p65 (p-NFκB; cat. no. 3033), anti-phospho-inhibitor of κBα (p-IκBα; cat. no. 2859), anti-phospho-extracellular signal-regulated kinase (p-ERK; cat. no. 4370T), anti-interleukin (IL)6 (cat. no. 12912), anti-IL-1β (cat. no. 12703S), anti-phospho-phosphatidylinositol 3-kinase (p-PI3K; cat. no. 4228S), and anti-glyceraldehyde 3-phosphate dehydrogenase (GAPDH; cat. no. 2118).

### Myofilament Phosphorylation

Myofilament phosphorylation levels were analyzed to examine their responses to chronic pressure overload. Myofilaments were extracted as previously described, with modifications (19–21). The hearts were homogenized in an ice-cold cell lysis buffer comprising 60 mM KCl, 30 mM imidazole (pH 7.0), 2 mM MgCl_2_, and proteinase inhibitors, and then centrifuged at 12,000 × *g* at 4°C for 15 min. Pellets were extracted with the same ice-cold lysis buffer containing 1% Triton X-100 and centrifuged at 1,100 × *g* to obtain myofilaments. Phosphorylation levels of myofilaments [myosin binding protein C (MyBP-C), desmin, cardiac troponin T (cTnT), tropomyosin, cardiac troponin I (cTnI), and myosin light chain 2 (MLC2)] were measured using Pro-Q Diamond Phosphoprotein Gel Stain according to the manufacturer's instructions (Molecular Probes, USA). Briefly, myofilaments (60 μg) were resolved by SDS-PAGE on 12% gels. After fixation with 50% methanol/10% acetic acid for 30 min twice, the gels were incubated with Pro-Q Diamond solution for 90 min. Phosphorylated bands were visualized using a Gel Doc XR+ gel documentation system (Bio-Rad Laboratories Ltd., USA). Total protein was stained with Coomassie brilliant blue (cat. no. 112553; Sigma-Aldrich, USA), and actin bands were used as loading controls.

### Statistical Analysis

All experimental data are presented as means ± standard errors of the means (SEMs). Raw data of the sham and TAC groups were input into GraphPad Prism 8.0 (GraphPad Software, USA) for statistics analysis. Unpaired *t*-tests and a two-sided *P*-value with a confidence level of 95% were used to determine the differences between the groups. *P* < 0.05 was considered significant. The graphs showing each individual data point were presented.

## Results

### Left Ventricles Display Concentric Hypertrophy and Then Dilation After DAC Surgery, With Preserved EF

In this study, we developed a surgical procedure to induce an HF model in Tibetan minipigs (Figure 1). Several key steps were performed, including localization of the descending aorta, analysis of the degree of aortic narrowing, and pressure measurements. One animal in each group did not have stable heart rates during the recording of echocardiography, so these two animals were excluded from experiments. Transmitral Doppler showed that the E: A ratio and the left atrial diameter were significantly higher in the DAC group than in the sham group. In addition, compared with the sham group, serum cTnI, CK, and LDH levels were significantly increased in the DAC group at week 12 (Table 2). These changes indicate diastolic dysfunction of the left ventricles.

**Table 2:** Indices of heart failure of the sham and DAC groups.

**Groups**		**Sham**	**DAC**
**Transmitral Doppler**			
	E (m/s)	0.58 ± 0.02	0.67 ± 0.07
	A (m/s)	0.48 ± 0.01	0.27 ± 0.03^***^
	E/A	1.22 ± 0.05	2.49 ± 0.14^***^
**Left atrial size**			
	LA (mm)	34.05 ± 0.57	42.43 ± 0.81^***^
**Laboratory parameters**			
	cTnI (pg/mL)	149.10 ± 28.92	340.06 ± 56.32^*^
	CK (U/L)	366.80 ± 34.40	546.25 ± 48.58^*^
	LDH (U/L)	255.40 ± 10.00	373.25 ± 15.53^***^
	Creatinine (μmol/L)	90.00 ± 12.52	85.00 ± 5.31

*All data are presented as the means ± SEMs. Sham, the sham group; DAC, the descending aortic constriction group; E, the peak early filling velocity; A, the velocity at atrial contraction; E/A, ratio of early diastolic blood flow peak E and late diastolic peak A; BNP, brain natriuretic peptide; cTnI, cardiac troponin I; CK, creatine kinase; LDH, lactate dehydrogenase*.

* *P < 0.05*;

*** *P < 0.001 vs. the sham group*.

**Figure 2:**
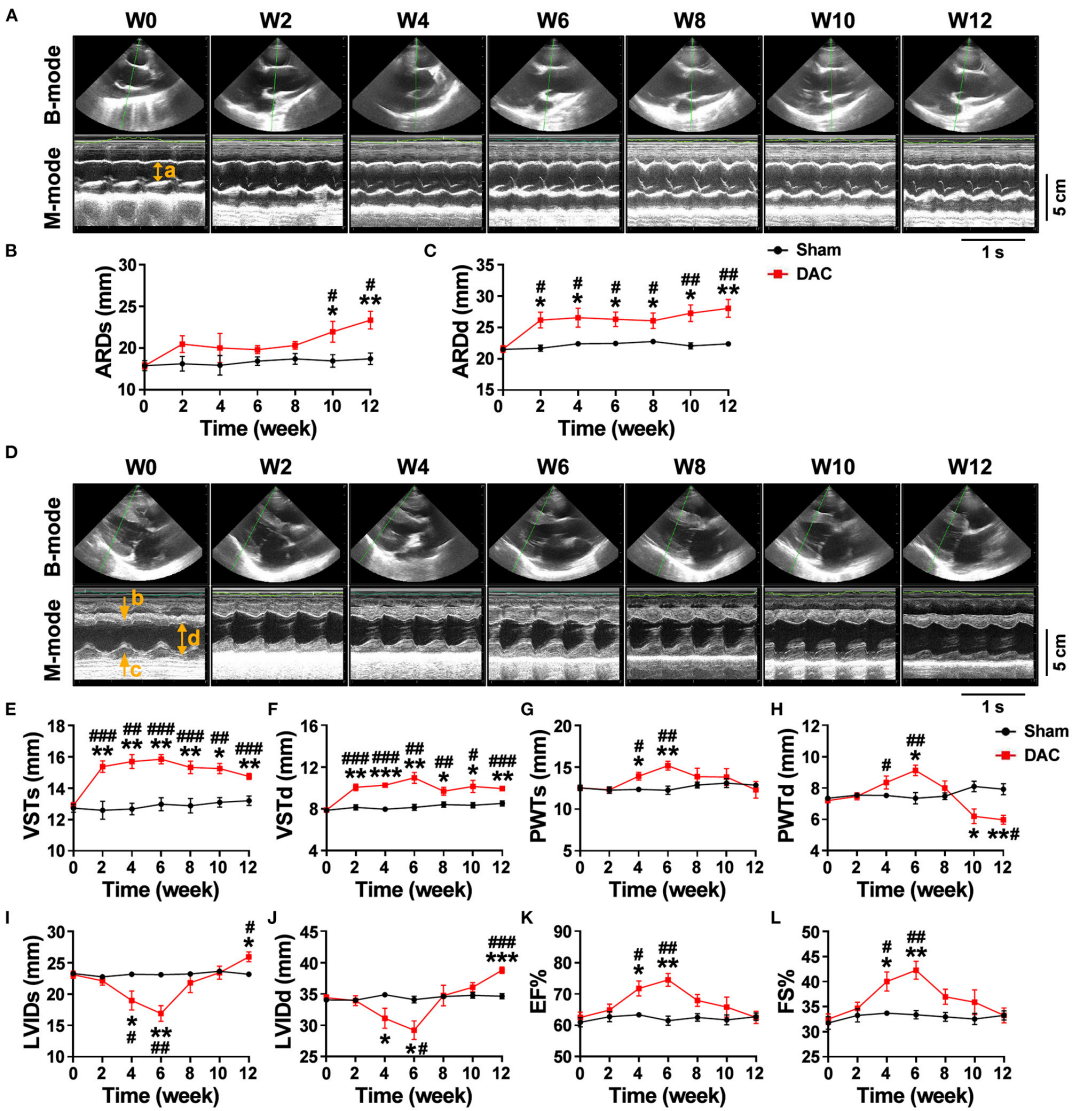
Echocardiography of the sham and DAC animals. B-mode and M-mode of the aortic root **(A)** and left ventricle **(D)** at weeks 0, 2, 4, 6, 8, 10, and 12. Following DAC surgery, the diameters of aortic roots at systole (ARDs) and diastole (ARDd) were increased following DAC **(B,C)**. The thicknesses of the intraventricular septum at systole (VSTs) and diastole (VSTd) and were significantly increased over time **(E,F)**. The thickness of posterior wall at systole (PWTs) and diastole (PWTd) in the DAC group were gradually increased after week 2, but decreased after week 8 **(G,H)**. Changes in the left ventricular internal dimension at systole (LVIDs) and diastole (LVIDd) displayed a pattern with a “V” shape over time after surgery **(I,J)**. The left ventricular ejection fraction (EF) and fractional shortening (FS) in the DAC groups were preserved, compared with those of the sham groups at the corresponding time points **(K,L)**. ARD (a), VST (b), PWT (c), and LVID (d) were indicated in M mode images of **(A)** and **(D)**. *n* = 4 minipigs per group. All data are presented as the means ± SEMs. *P < 0.05, **P < 0.01, ***P < 0.001 vs. the sham group; ^#^*P* < 0.05, ^##^*P* < 0.01, ^###^*P* < 0.001 vs. the week 0.

Echocardiography showed that the aortic roots of minipigs gradually expanded over time after surgery (Figure 2A). The ARDd was also significantly larger in the DAC group than in the sham group at weeks 2, 4, 6, 8, 10, and 12 following DAC surgery (Figures 2B,C, Table 3). Left ventricles were remodeled after the DAC surgery (Figure 2D). Specifically, VST values were significantly altered at each of the post-surgical time points. Increases in VSTs (Figure 2E) and VSTd (Figure 2F) started at week 2, while changes in the PWTs and PWTd started at week 4. VSTs and VSTd hypertrophy remained at week 4, 6, and 8, but posterior wall hypertrophy was not observed at these time points. After 10 and 12 weeks, the PWTd was significantly decreased in the DAC group compared with the sham group (Figures 2G,H). The results were summarized in Table 3.

**Table 3:** Echocardiographic analysis of the sham and DAC groups.

**Time**		**Week 0**	**Week 2**	**Week 4**	**Week 6**	**Week 8**	**Week 10**	**Week 12**
ARDd (mm)	Sham	21.5 ± 0.3	21.7 ± 0.5	22.4 ± 0.3	22.5 ± 0.4	22.8 ± 0.1	22.1 ± 0.5	22.4 ± 0.3
	DAC	21.6 ± 0.5	26.2 ± 1.3^*^^#^	26.6 ± 1.5^*^^#^	26.3 ± 1.2^*^^#^	26.1 ± 1.2^*^^#^	27.3 ± 1.3^*^^##^	28.1 ± 1.4^**^^##^
ARDs (mm)	Sham	17.9 ± 0.6	18.1 ± 0.9	18.0 ± 1.2	18.5 ± 0.5	18.7 ± 0.6	18.5 ± 0.8	18.7 ± 0.7
	DAC	17.9 ± 0.4	20.5 ± 1.0	20.0 ± 1.7	19.8 ± 0.5	20.3 ± 0.5	22.0 ± 1.3^*^^#^	23.4 ± 1.1^**^^#^
VSTs (mm)	Sham	12.7 ± 0.3	12.6 ± 0.6	12.7 ± 0.4	13.0 ± 0.4	12.9 ± 0.5	13.1 ± 0.3	13.2 ± 0.3
	DAC	12.9 ± 0.2	15.4 ± 0.4^**^^###^	15.7 ± 0.4^**^^##^	15.9 ± 0.3^**^^###^	15.3 ± 0.4^**^^###^	15.3 ± 0.3^*^^##^	14.8 ± 0.2^**^^###^
VSTd (mm)	Sham	7.9 ± 0.2	8.2 ± 0.2	8.0 ± 0.1	8.2 ± 0.3	8.4 ± 0.2	8.4 ± 0.3	8.5 ± 0.2
	DAC	7.9 ± 0.1	10.1 ± 0.3^**^^###^	10.3 ± 0.2^***^^###^	11.0 ± 0.5^**^^##^	9.7 ± 0.4^*^^##^	10.2 ± 0.6^*^^#^	10.0 ± 0.2^**^^###^
PWTs (mm)	Sham	12.5 ± 0.3	12.3 ± 0.4	12.4 ± 0.2	12.3 ± 0.5	12.9 ± 0.3	13.1 ± 0.5	12.9 ± 0.3
	DAC	12.5 ± 0.2	12.3 ± 0.2	13.9 ± 0.5^*^^#^	15.2 ± 0.5^**^^##^	13.9 ± 1.0	13.8 ± 1.0	12.3 ± 1.0
PWTd (mm)	Sham	7.4 ± 0.1	7.6 ± 0.1	7.5 ± 0.1	7.4 ± 0.4	7.5 ± 0.2	8.1 ± 0.3	7.9 ± 0.4
	DAC	7.2 ± 0.2	7.5 ± 0.2	8.4 ± 0.4^#^	9.1 ± 0.3^*^^##^	8.0 ± 0.5	6.2 ± 0.5^*^	6.0 ± 0.3^**^^#^
LVIDs (mm)	Sham	23.3 ± 0.2	22.8 ± 0.4	23.2 ± 0.4	23.1 ± 0.4	23.2 ± 0.2	23.6 ± 0.4	23.2 ± 0.3
	DAC	23.1 ± 0.6	22.1 ± 0.7	19.0 ± 1.5^*^^#^	16.9 ± 1.2^**^^##^	21.8 ± 1.6	23.4 ± 1.0	25.9 ± 0.8^*^^#^
LVIDd (mm)	Sham	34.1 ± 0.3	34.0 ± 0.4	34.9 ± 0.4	34.1 ± 0.5	34.6 ± 0.4	34.8 ± 0.5	34.7 ± 0.4
	DAC	34.4 ± 0.5	33.9 ± 0.8	31.1 ± 1.6^*^	29.2 ± 1.5^*^^#^	34.8 ± 1.7	36.1 ± 0.7	38.8 ± 0.5^***^^###^
EF (%)	Sham	61.0 ± 1.5	62.8 ± 1.6	63.4 ± 0.7	61.5 ± 1.4	62.5 ± 1.2	61.8 ± 1.6	62.8 ± 1.2
	DAC	62.5 ± 1.7	64.9 ± 1.9	71.8 ± 2.4^*^^#^	74.5 ± 2.0^**^^##^	68.0 ± 1.9	65.8 ± 3.2	62.5 ± 1.9
FS (%)	Sham	31.8 ± 1.3	33.3 ± 1.3	33.7 ± 0.5	33.4 ± 0.8	33.0 ± 0.9	32.5 ± 1.1	33.3 ± 0.9
	DAC	32.5 ± 1.0	34.7 ± 1.2	40.0 ± 1.9^*^^#^	42.3 ± 1.8^**^^##^	37.0 ± 1.5	35.9 ± 2.5	33.3 ± 1.5

*All data are presented as the means ± SEMs. DAC, descending aortic constriction group; ARDs and ARDd, aortic root diameter at the end of systole and diastole, respectively; VSTs and VSTd, thickness of the ventricular septum at the end of systole and diastole, respectively; PWTs and PWTd, thickness of the posterior wall at the end of systole and diastole, respectively; LVIDs and LVIDd, left ventricular internal dimension at the end of systole and diastole, respectively; EF, left ventricular ejection fraction; FS, left ventricular fraction shortening*.

* *P < 0.05*;

** *P < 0.01*;

*** *P < 0.001 vs. the sham group*;

# *P < 0.05*;

## *P < 0.01*;

### *P < 0.001 vs. the week 0*.

Changes in the LVID displayed a V-shaped pattern over time after surgery (Figures 2I,J, Table 3). At week 2, the LVID at systole or diastole was decreased in DAC animals, with the smallest diameter at week 6. At week 10, the LVID started to enlarge, resulting in a significant difference compared with the LVID in sham animals at week 12. At the stage of ventricular hypertrophy in the DAC animals, EF and FS were increased, and at weeks 4 and 6, the increases were significantly different compared with the two indices in the pre-surgery group and the sham group at the corresponding time points. However, in dilated ventricles, the EF and FS were preserved in the DAC group compared with those observed in the sham group at the corresponding time points (Figures 2K,L, Table 3).

### DAC Induces Pulmonary and Cardiovascular Damage, Including Fibrosis, Inflammatory Cell Infiltration, and Cardiomyocyte Hypertrophy

**Figure 3:**
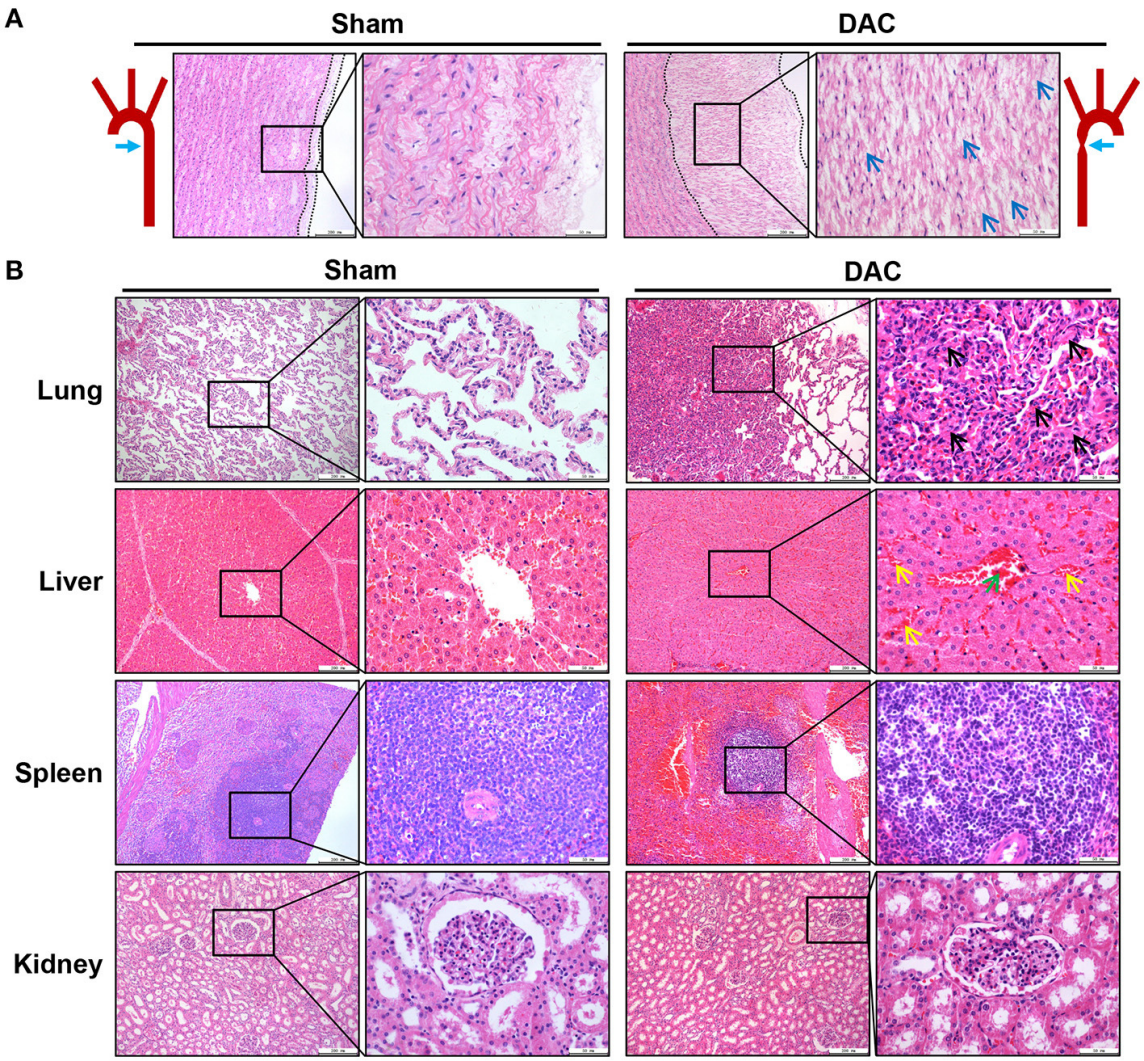
Pathological changes of the aorta **(A)** and main organs **(B)** of lungs, livers, spleens, and kidneys of the sham and DAC minipigs. In DAC animals at 12 weeks after surgery, the intima of the proximal aorta displayed hyperplasia, indicated at **(A)**. The collagen fibers in the subendothelial layer were indicated (blue arrows). The alveolar wall thickened (black arrows). In addition, hepatic tissues displayed hepatocellular oedema, congestion in the central vein (green arrows), and hepatic sinusoids (yellow arrows).

Pathological examination was performed on the aortas, atria, ventricles, lungs, livers, spleens, and kidneys of sham and DAC minipigs 12 weeks after surgery (Figures 3A,B). In DAC animals, the intima of the proximal aorta displayed hyperplasia, mainly manifested as an increase in collagen fibers in the subendothelial layer. The alveolar wall was thickened. In addition, hepatic tissues displayed hepatocellular edema, congestion in the central vein, and hepatic sinusoids. There were no obvious lesions caused by DAC in the kidneys or spleen.

**Figure 4:**
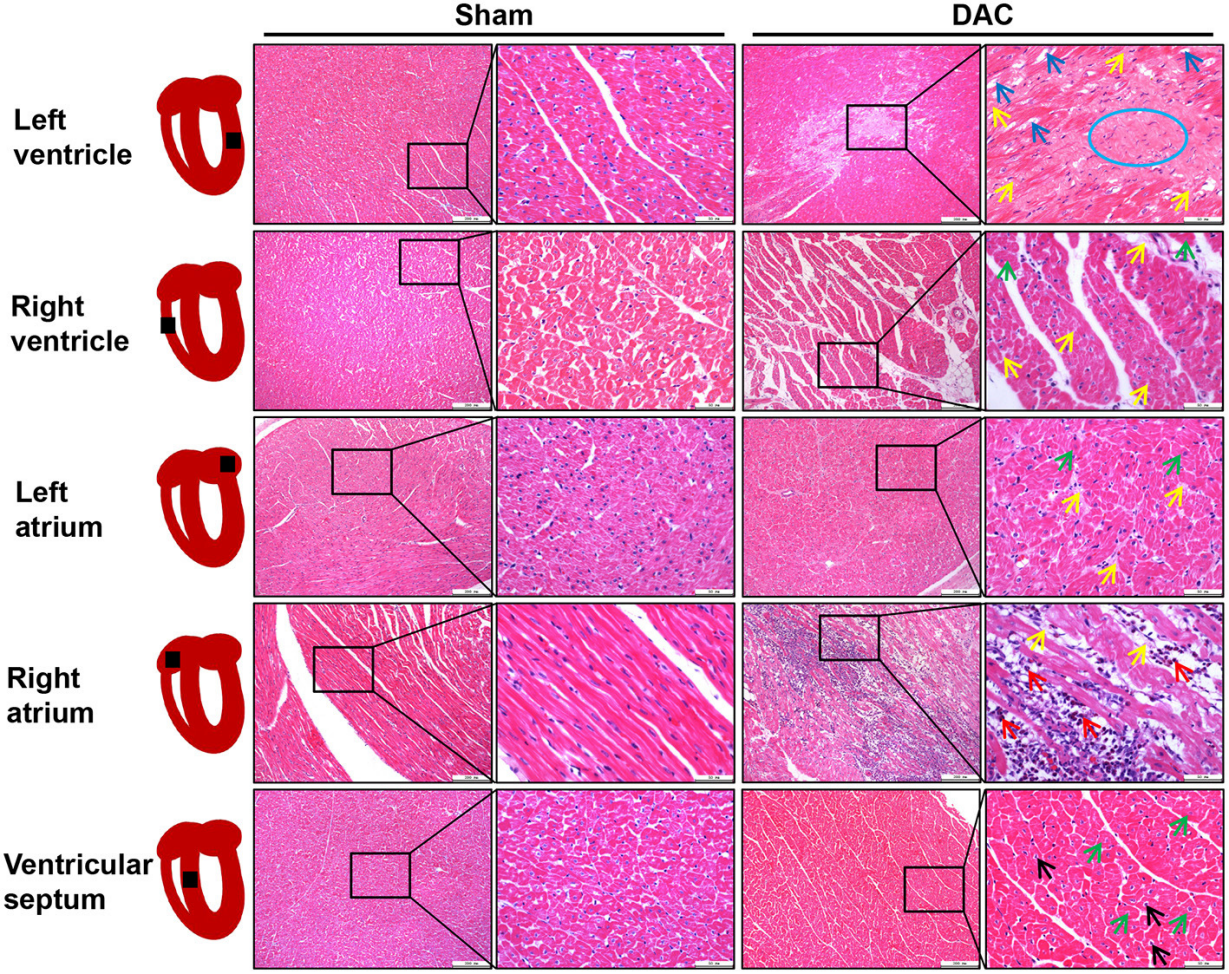
Tissue damage of atria, ventricles, and interventricular septum induced by DAC. There was widespread fibrosis (yellow arrows) in the myocardium of the left and right atria. Cardiomyocyte hypertrophy (green arrows) and eosinophil infiltration (red arrows) were observed in the right atrium. Cardiomyocyte hypertrophy (green arrows) and nuclear pyknosis (black arrows) were observed in the internal ventricular septum. Fibrosis (yellow arrows) was present in the left ventricular myocardium, accompanied by dissolved cardiomyocyte nuclei (blue arrows) and scar (blue circle) appearance. The right ventricle showed cardiomyocyte hypertrophy (green arrows) and interstitial fibrosis (yellow arrows).

Next, the ventricles, atria, and ventricular septa were examined (Figure 4). There was widespread fibrosis in the myocardium of the left and right atria. Eosinophil infiltration was observed in the right atrium. Cardiomyocyte hypertrophy and nuclear pyknosis were observed in the internal ventricular septum. Moreover, fibrosis was widely present in the left ventricular myocardium, accompanied by dissolved cardiomyocyte nuclei and scarring. The right ventricle revealed cardiomyocyte hypertrophy and interstitial fibrosis. Overall, cardiac damage induced by DAC was characterized by fibrosis, eosinophil infiltration, and cardiomyocyte hypertrophy.

### The Myocardium of the HFpEF Model Exhibits Evidence of Fibrosis

**Figure 5:**
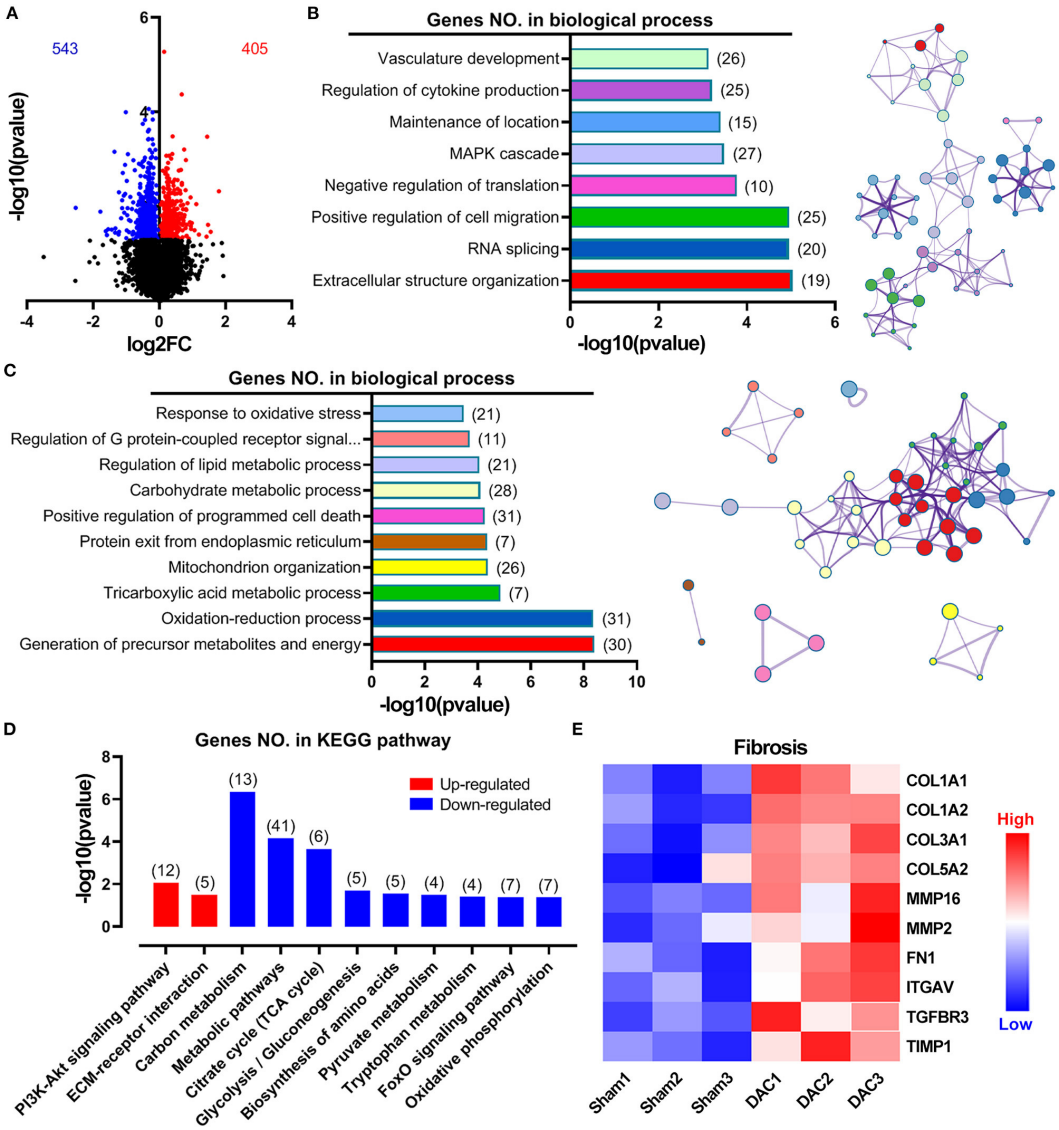
Overall gene expression profiles in sham and DAC animals. Gene enrichment analyses suggested that heart cells in HFpEF suffer inflammatory stress, oxidative stress, remodeling, proliferation, apoptosis, and metabolic disorders. Specifically, 543 genes were downregulated, whereas 405 genes were upregulated in the DAC myocardium compared with that in the sham myocardium **(A)**. Biological process enrichment analysis identified eight upregulated biological processes **(B)** and 10 downregulated biological processes **(C)**. KEGG pathway analysis showed that two pathways were upregulated and night pathways were downregulated **(D)**. In addition, 10 genes associated with fibrosis were seen in **(E)**. *n* = 3 minipigs per group.

Overall gene expression profiles demonstrated that 543 genes were downregulated and 405 genes were upregulated in the DAC myocardium compared with in the sham myocardium (Figure 5A). Biological process enrichment analysis identified eight upregulated biological processes, including regulation of vasculature development (26 genes), regulation of cytokine production (25 genes), maintenance of location (15 genes), mitogen-activated protein kinase cascade (27 genes), negative regulation of translation (10 genes), positive regulation of cell migration (25 genes), RNA splicing (20 genes), and extracellular structure organization (19 genes; Figure 5B). In addition, we identified 10 downregulated biological processes, including response to oxidative stress (21 genes), regulation of G-protein-coupled receptor signaling (11 genes), regulation of lipid metabolic process (21 genes), carbohydrate metabolic process (28 genes), positive regulation of programmed cell death (31 genes), protein exit from the endoplasmic reticulum (7 genes), mitochondrion organization (26 genes), tricarboxylic acid metabolic process (7 genes), oxidation-reduction process (31 genes), and generation of precursor metabolites and energy (30 genes; Figure 5C). KEGG pathway analysis showed that two pathways were upregulated, including the PI3K/Akt signaling pathway and extracellular matrix/receptor interactions, and 9 pathways were down regulated (Figure 5D). Among these downregulated pathways, 7 pathways were involved in cellular metabolisms, including the carbon metabolism, metabolic pathways, citrate cycle, glycolysis/gluconeogenesis, biosynthesis of amino acids, pyruvate metabolism, and tryptophan metabolism. Thus, enrichment analyses suggested that heart cells in HFpEF suffer from inflammatory and oxidative stress and have altered remodeling, proliferation, apoptosis, and metabolic signaling. Furthermore, cluster analysis identified 10 genes associated with fibrosis, including *COL1A1, COL1A2, COL3A1, COL5A2, MMP16, MMP2, Fibronectin 1, Integrin Subunit Alpha V, Transforming Growth Factor Beta Receptor 3*, and *TIMP1* (Figure 5E). These findings suggest that fibrosis occurs in the myocardium of minipigs with HFpEF.

### Decreased Phosphorylation of Cardiac Myofilaments Is Associated With Cardiac Remodeling and Diastolic Dysfunction in HFpEF

**Figure 6:**
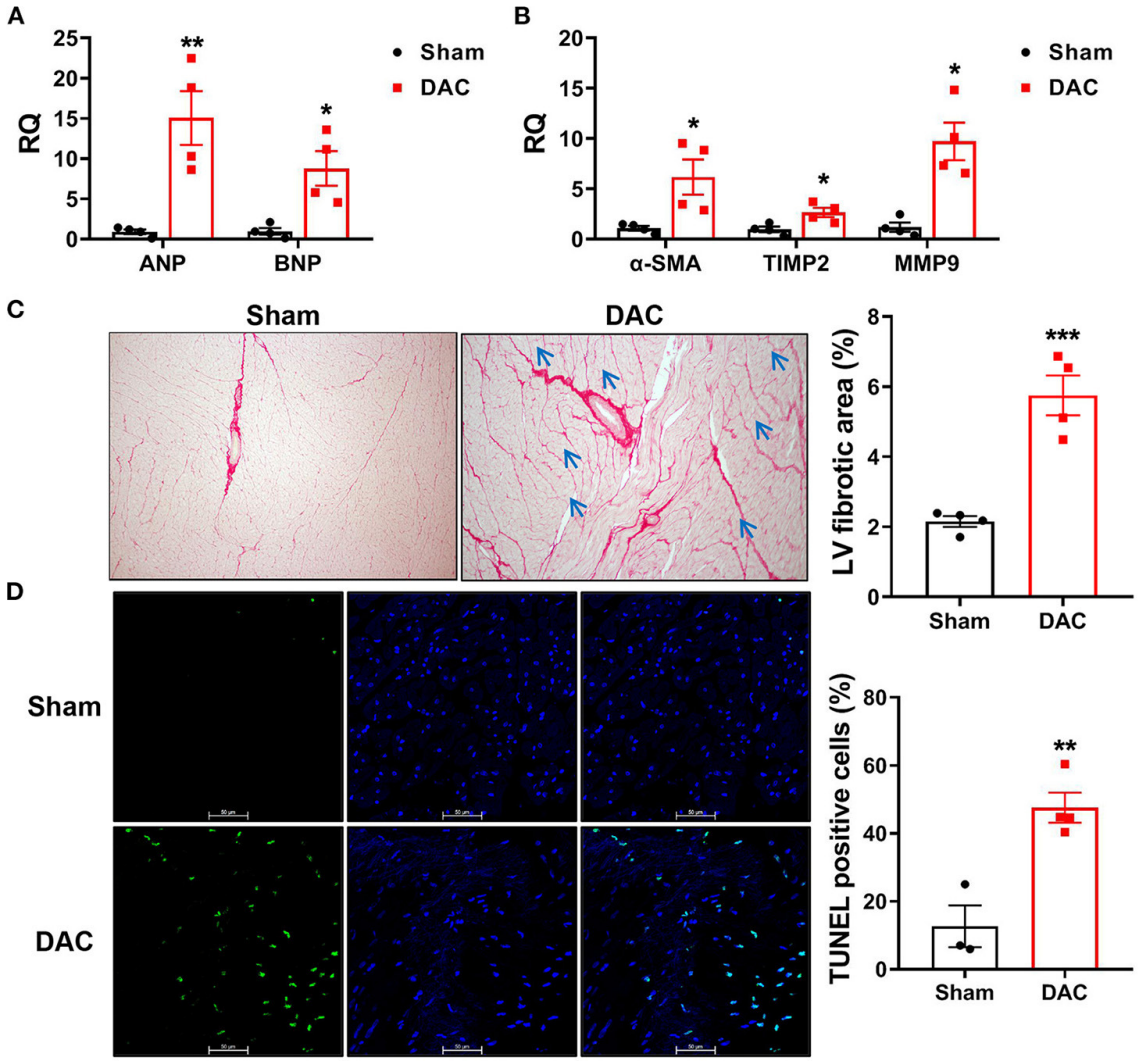
Biomarkers of heart failure, fibrotic area, and apoptosis in the left ventricles. The cardiac injuries at the cellular and molecular levels were seen of the left ventricles of the minipigs with HFpEF. The expression levels of ANP and BNP **(A)** and α-SMA, TIMP2, MMP9 **(B)** were significantly higher in the DAC group than those in the sham group. *n* = 4 minipigs per group. Sirius Red staining showed the fibrotic area in the DAC group was significantly greater than that in the sham group **(C)**, and the cardiomyocytes in the DAC group displayed hypertrophy (blue arrows), and apoptosis **(D)** was observed in the myocardium and epicardium of HFpEF. *n* = 3–4 minipigs per group. All data are presented as the means ± SEMs. *P < 0.05, **P < 0.01, ***P < 0.001 vs. the sham group.

Cardiac injuries in the left ventricles of the minipigs with HFpEF were observed at the cellular and molecular levels. RT-qPCR showed that expression levels of the heart failure markers (ANP and BNP) and the fibrotic markers (α-SMA, TIMP2, and MMP9) were significantly higher in the DAC group than in the sham group (Figures 6A,B). Sirius Red staining revealed that the fibrotic area was significantly greater in the DAC group than in the sham group (Figure 6C). Additionally, the cardiomyocytes displayed hypertrophy, and apoptosis was observed in the myocardium and epicardium of the DAC group (Figures 6C,D).

**Figure 7:**
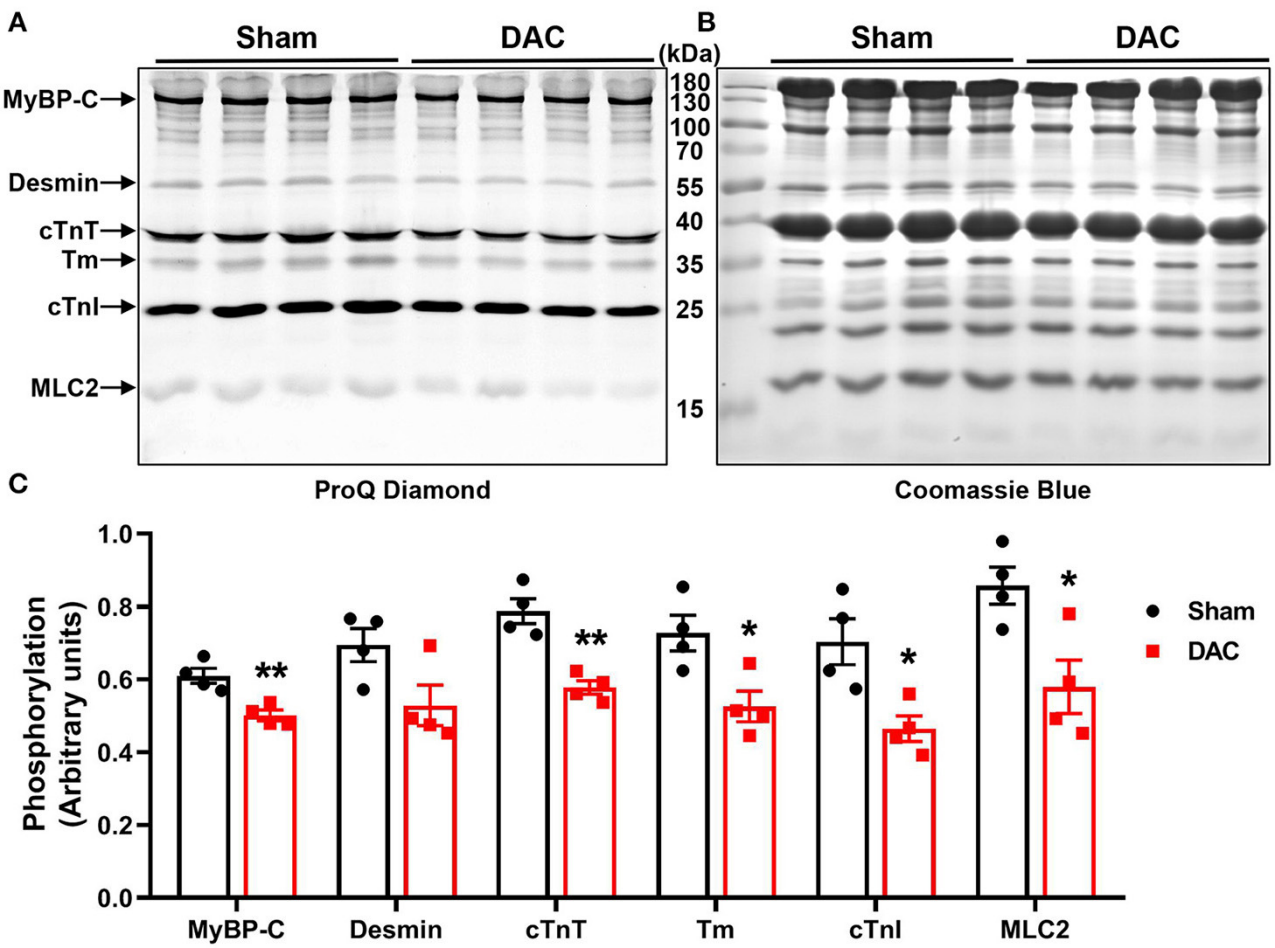
Phosphorylation of contractile proteins. Phosphorylation levels of MyBP-C, desmin, cTnT, tropomyosin (Tm), cTnI, and MLC2 were significantly suppressed **(A,C)** in minipigs with HFpEF induced by chronic pressure overload. **(A)** Pro-Q phosphorylation staining image, each band represents an individual animal. *n* = 4 minipigs per group. *P < 0.05, **P < 0.01 vs. the sham group.

Upon examining alterations in myofilament phosphoproteins that regulate cardiac diastolic function, we found that the phosphorylation levels of myofilament proteins (MyBP-C, desmin, cTnT, tropomyosin, cTnI, and MLC2) were significantly suppressed in minipigs with HFpEF induced by chronic pressure overload compared to sham pigs (Figure 7). Study has shown that myofilament phosphorylation is essential for normal diastolic function in HFpEF hearts (22), and in the end stages of HF, aged dogs with HFpEF display lower levels of myofilament phosphorylation than healthy dogs (23). This suggested impaired diastolic function in the heart, and indicated that cardiomyocyte hypertrophy, apoptosis, and fibrosis contribute to cardiac remodeling in HFpEF.

### Activation of Fibrotic and Inflammatory Pathways in Chronic Pressure Overload-Induced HFpEF

**Figure 8:**
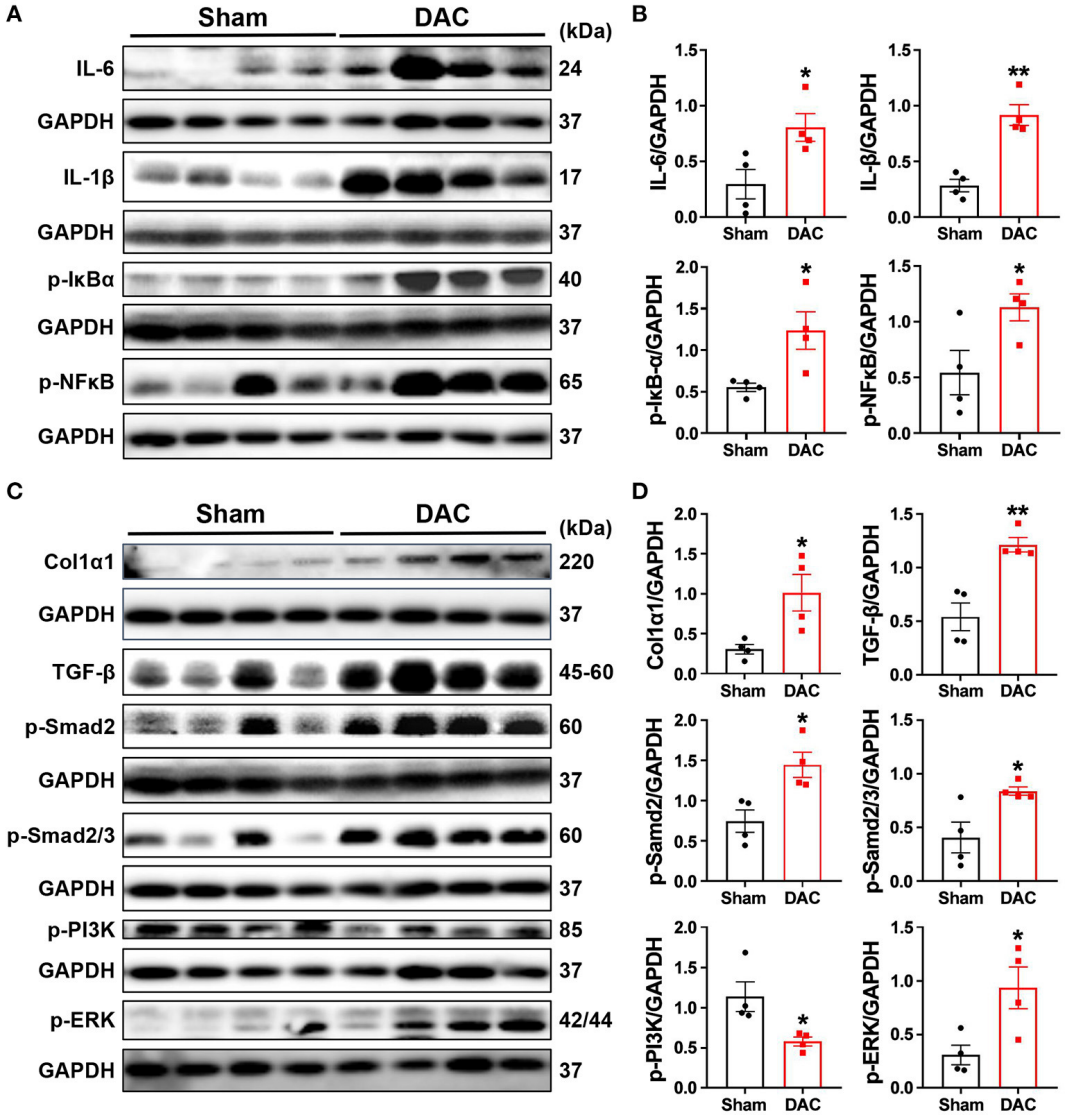
Activation of the inflammatory and fibrotic pathways. This study demonstrates that myocardial fibrosis is associated with activation of inflammatory pathways as the evident elevation of p-IκBα, p-NFκB, IL-6, and IL-1β in the DAC myocardium **(A,B)** and significant increases in the levels of fibroblast-secreted TGF-β1, the downstream effectors p-Smad2 and p-Smad2/3, and p-PI3K and p-ERK linking to cellular proliferation following DAC **(C,D)**. *n* = 4 minipigs per group. Each band represent an individual animal. All data are presented as the means ± SEMs. *P < 0.05, **P < 0.01 vs. the sham group.

Both pathological examination and GSEA suggested that inflammatory responses were critical events in the impairment of heart cells. Secretion of cytokines upon mechanical overload is known to trigger the activation of fibroblasts. Therefore, we investigated inflammatory signaling pathways and found that DAC elevated the levels of p-IκBα, p-NFκB, IL6, and IL-1β in the minipig myocardium (Figures 8A,B). These results suggested that activation of fibrotic signaling pathways promotes the differentiation of myofibroblasts in hearts with chronic pressure overload.

Next, we investigated whether fibrosis-associated molecular signals were activated in HFpEF. Importantly, we observed significant increases in the levels of fibroblast-secreted TGF-β, its downstream effectors p-SMAD2 and p-SMAD2/3, and proteins associated with cellular proliferation, including p-PI3K and p-ERK, following DAC (Figures 8C,D). Additionally, transcriptional sequencing analysis showed increases in MMP2 and TIMP1 levels in HFpEF hearts compared with sham hearts, accompanied by increases in collagen I protein expression (Figures 8C,D). These findings explain the fibrosis observed in the myocardium of HFpEF.

## Discussion

**Figure 9:**
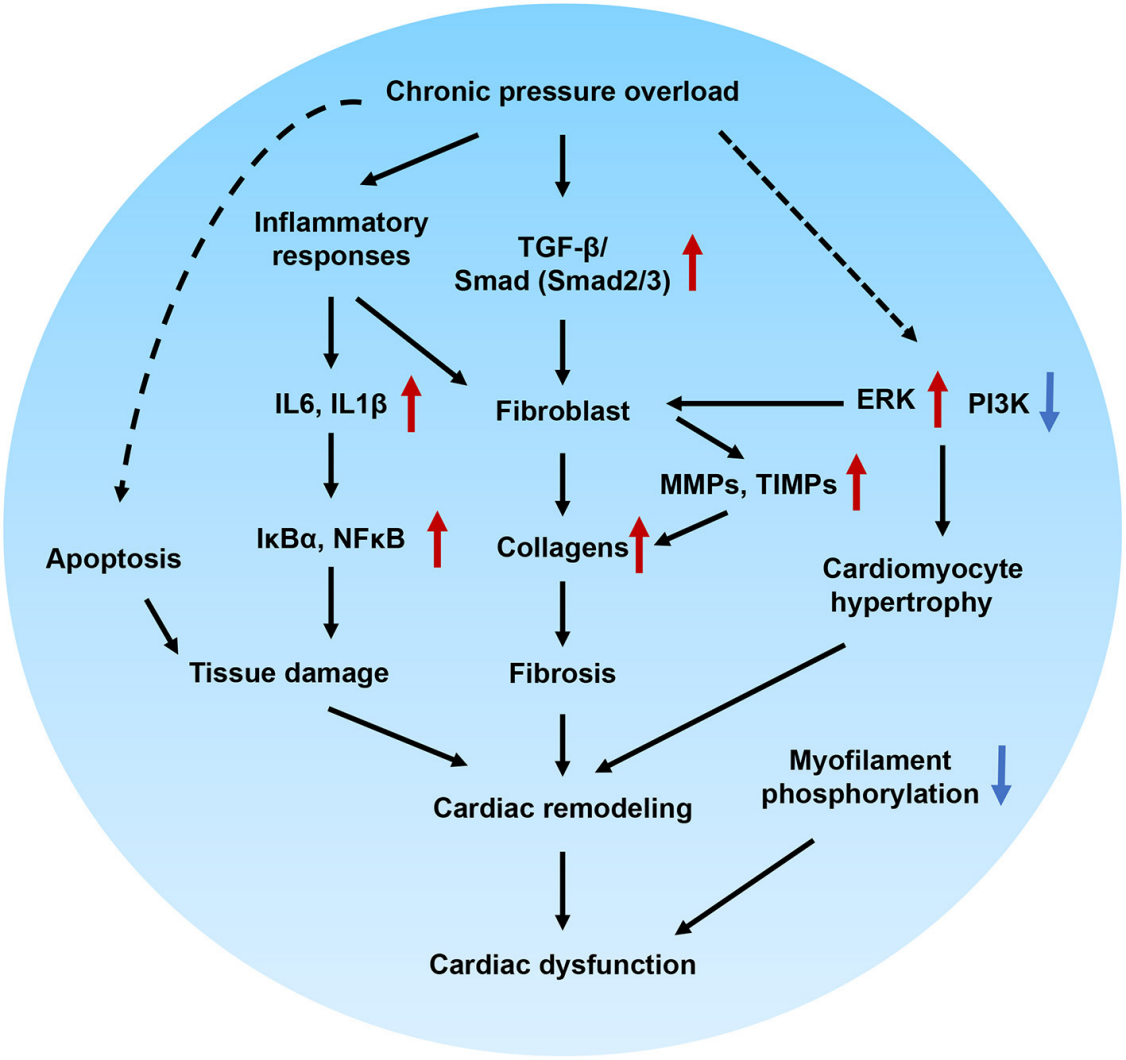
Proposed molecular mechanisms underlying cardiac remodeling and functional impairment in HFpEF. Chronic pressure overload induces inflammatory responses, which in turn activates cytokines and intracellular NFκB signaling and associated with tissue damage. In addition, inflammatory stimuli also involved in the fibrotic signaling. On the other hand, chronic pressure overload activates TGF-β/Smad signaling pathways, which together with inflammatory stimuli, PI3K/ERK signaling promote proliferation and differentiation of fibroblast. Furthermore, MMPs, TIMPs and several other signals regulates the production of collagens. The tissue damage, fibrosis, cardiomocyte hypertrophy, and myofilament dephosphorylation contribute to cardiac remodeling and diastolic dysfunction.

In this study, we successfully developed a novel HFpEF model using DAC-induced chronic pressure overload in minipigs. This model was characterized by cardiac remodeling, fibrosis, tissue damage, and impaired cellular signal transduction. Echocardiography was performed to trace the aortic and ventricular morphology and cardiac contractile function over 12 weeks, demonstrating the pattern of HFpEF disease progression. In our further investigation of the mechanisms underlying the pathological and cellular responses, the fibrotic signaling pathways in the myocardium of HFpEF were explored. Based on this, we can propose a potential regulatory mechanism underlying cardiac remodeling and functional impairment in HFpEF (Figure 9). Briefly, chronic pressure overload induces inflammatory responses, which in turn activate cytokines and intracellular NFκB signaling, resulting in tissue damage. In addition, inflammatory stimuli are also involved in fibrotic signaling. On the other hand, chronic pressure overload activates TGF-β/SMAD signaling, which together with inflammatory stimuli and PI3K/ERK activation, promotes proliferation and fibroblast differentiation. Furthermore, MMPs, TIMPs, and several other signals increase collagen production. The resultant tissue damage, fibrosis, cardiomyocyte hypertrophy, and myofilament dephosphorylation all contribute to cardiac remodeling and dysfunction. This is the first study to use DAC to model HFpEF, establishing a powerful tool for modeling this condition.

### Assessment of Pathological and Cellular Responses of HFpEF Model

HFpEF is a heterogeneous cardiac phenotype with many patterns of cardiac remodeling. These structural phenotypes include normal cardiac tissue, concentric remodeling, concentric hypertrophy, and eccentric hypertrophy (24). We found that constriction at the descending aorta trunk was suitable for linear studies of structural and functional changes following chronic pressure overload. During the 12-week observation period, we detected concentric hypertrophy, hypertrophy with normal LVID, and left ventricle dilation with normal interventricular septum thickness, suggesting that our model mimics the conditions in human HFpEF. In addition, the results of our pathological examination were consistent with other studies describing cardiomyocyte hypertrophy (25). Here, we conclude that echocardiography is the appropriate imaging tool to evaluate cardiac structure and function in the laboratory study of large animal HFpEF models. Furthermore, consistent with the structural findings from echocardiography, gene ontology enrichment analysis demonstrated the upregulation of several biological processes associated with cardiac remodeling, including vasculature development, positive regulation of cell migration, and extracellular structure.

Regarding the selection of constriction sites for inducing pressure overload, the aortic root, and ascending, transverse, and descending trunks have been used in small and large animal models (13, 16, 18, 26). The ascending or transverse trunks are more favorable in small animal HF models. These sites are approachable by either using a medical needle to determine the constriction degree or placing a ring of fixed size (13, 16). In large animals, the internal aortic diameters are comparable with those of humans. With the aid of a catheter and angiogram technology, researchers can deliver medical stents (26) or inflatable cuffs (18) to any fragments of the aorta to execute vessel constriction. Thus, compared with small animals, aortic constriction sites are less restricted by thoracic anatomy in large animals. However, the high cost of angiography machines has hindered the use of catheters. In this study, we adopted the thoracotomy used in small animals, but applied the incision on the left chest. This strategy avoids sternum damage while clearly exposing the aortic descending trunk. More importantly, this large animal HFpEF model can be generated without the need for expensive instrument. Besides the descending aorta, banding at the ascending aorta was performed by two independent groups (27–29). In these cases, the third intercostal space was widened to locate the ascending aortic trunk (27).

Several methods have been used to develop HFpEF pig models. Deoxycorticosterone acetate (DOCA) combined with a Western diet was used to induce hypertension and hyperlipidemia in Landrace pigs for 12 weeks (22), and it was later shown that DOCA alone can induce the development of early stage HFpEF in pigs (30). A combination of a Western diet and pressure overload has been used to study HFpEF in rodent models, and this approach was recently introduced in a pig model (31). Compared with these methods, our model has the advantage of introducing chronic pressure overload as a condition of hypertension, one of the most common chronic diseases related to HFpEF.

Metabolic KEGG pathway analysis showed that the DEGs between healthy and HFpEF myocardia were involved in citrate cycle suppression, glycolysis, amino acid biosynthesis, and pyruvate metabolism, suggesting that metabolic changes are crucial in HFpEF. Our findings are consistent with a study in a murine HFpEF model of metabolic disorder, in which researchers found that β-hydroxybutyrate was an effective treatment, as it targets the acetyl-CoA pool and mitochondrial acetylation (32). Furthermore, a clinical study including 46 patients with new-onset HFpEF and 75 patients with new-onset HFrEF revealed 11 plasma metabolites that differed between the two groups, and proposed that the hearts of patients with HFpEF tended to show increased rates of fibrosis, oxidative stress, and inflammation compared with those of patients with HFrEF (33). Together, these results suggested that metabolic pathways could be effective targets for HFpEF therapy.

### Cardiac Fibrosis Is a Key Response to Chronic Pressure Overload-Induced HFpEF

Fibrosis is a cellular response to harmful stimuli such as mechanical stress, and is mainly caused by the excessive deposition of extracellular matrix proteins. The key signal transduction pathways regulating the development of fibrous connective tissues include TGF-β signaling and growth-promoting PI3K signaling pathways. In the current study, we found that the TGF-β expression level was upregulated by chronic pressure overload, as were the phosphorylation levels of SMAD2 and SMAD3. However, we did not identify the distinct roles of each cellular signaling protein in the development of HF in this study. In pressure overload, deletion of the genes encoding TGF-β receptor and SMAD3 reduces the fibrotic response; additionally, deletion of the TGF-β receptor reduces the hypertrophic response, whereas deletion of *SMAD2/3* reduces cellular expansion (34). In future studies, identifying the different roles of these proteins will help to explain how hearts respond to biomechanical stress. In response to stimulation with TGF-β, cardiac fibroblasts switch to a myofibroblast phenotype expressing α-SMA, which is increased in pressure overload hearts displaying fibrosis (35). Consistent with their findings, we observed elevated gene expression of myofibroblast-derived α-SMA and protein expression of collagen I in the myocardium of HFpEF animals. Moreover, we found downregulated PI3K and upregulated ERK in the fibrotic myocardium. It has been known that PI3K and ERK signaling pathways are involved in the regulation of fibrosis. For example, suppression of α-SMA expression in cardiac fibroblasts is dependent upon PI3K signaling (36), and the suppression of cardiac fibrosis by chemical compounds (such as gentisic acid) occurs through the ERK signaling pathway (37). However, the regulatory mechanisms by which pathways affect the progression of HFpEF needs further investigation.

Enzymes such as MMP2, MMP9, and their inhibitors are known to regulate extracellular matrix turnover and induce cardiac fibrosis. Bergman et al. reported that MMP2 transgenics exhibit significant increases in interstitial and perivascular collagen in the heart, accompanied by marked ventricular remodeling (38). Moreover, deletion of *MMP2* or *TIMP1* minimizes fibrosis in pressure-overloaded mouse hearts (39). Notably, elevation of serum TIMP1 levels in the hypertensive heart is associated with left ventricular diastolic dysfunction and correlated with the elevation of other fibrotic markers, including plasma procollagen type I carboxy-terminal pro-peptide and the carboxy-terminal telopeptide of collagen type I (40). Similarly, in this study, we found that the expression levels of MMP2 and its inhibitor TIMP2, and MMP9 and its inhibitor TIMP1 were upregulated in the myocardium of HFpEF. These findings suggested that the porcine HFpEF model is an effective tool for studying the extracellular matrix turnover-dependent fibrotic mechanisms underlying this disease. However, we did not measure the protein expression levels of these enzymes and their inhibitors. Further studies are needed to elucidate the mechanisms underlying HFpEF.

Regarding the expression of MMP inhibitors, clinical studies have demonstrated that elevated TIMP1 is associated with fibrosis and cardiac remodeling in patients with hypertension; in contrast, in mechanistic studies in animal models, the inhibition of TIMP1 or TIMP2 effectively suppresses cardiac fibrosis and remodeling (39, 41). These differences may be due to analysis of samples from different stages (i.e., the compensatory and decompensatory stages) of cardiac remodeling. Importantly, in end-stage cardiomyopathy, higher levels of TIMP1 and TIMP2 are associated with cardiac fibrosis (42). However, further studies are needed to investigate the regulation of MMPs and their inhibitors.

### The Impairment of Diastolic Function in HFpEF

Although HFpEF has discrete pathophysiologic phenotypes, diastolic dysfunction is among its key features. Consistent with observations in clinical and experimental studies (43, 44), several key changes indicating diastolic dysfunction exist in our HFpEF model. These include changes in the E: A ratio, left atrial size, and serum cTnI, CK, and LDH levels. However, the mechanisms underlying diastolic dysfunction in the development of HFpEF are still unclear. Fibrosis and inflammation can have marked effects on diastolic function. In addition to profibrotic signaling triggered by TGF-β, inflammatory cytokines also promote myofibroblast differentiation (45). Clinical research has shown that the inflammatory biomarkers IL6 and tumor necrosis factor α are strongly associated with HFpEF in older adults (46) and in HFpEF animals (25). Furthermore, treatment with anti-chemoattractant protein inhibits macrophage accumulation and fibroblast proliferation, and is associated with improved remodeling and diastolic dysfunction (47). Here, we have shown that IL6, IL-1β, NFκB, and IκBα are significantly upregulated in the fibrotic myocardium of minipigs with diastolic dysfunction. Gene ontology enrichment analysis also demonstrated effects on the regulation of cytokine production. Together with our other findings, we conclude that cardiomyocyte hypertrophy, inflammatory responses, and apoptosis synergistically determine cardiac diastolic dysfunction in this porcine HFpEF model.

Furthermore, the phosphorylation of myofilament proteins is essential for normal diastolic function. In a previous study, Rosas et al. reported that MyBP-C phosphorylation benefits relaxation, whereas decreased phosphorylation of this myofilament protein disrupts diastolic function, a characteristic of HFpEF (48). In this study, we found that the phosphorylation levels of myofilament proteins were significantly lower in the hearts of HFpEF animals than in sham hearts, consistent with a study in a canine HFpEF model showing reduced phosphorylation of MyBP-C, TnT, TnI, and MLC2 (23). Hamdani et al. (23) further found that the phosphorylation levels of several titin sites were decreased, accompanied by corresponding changes in the expression levels of protein kinases and phosphatases. Our previous study revealed that myofilament phosphorylation is sensitive to all cardiac stresses (19, 21). However, myofilament phosphorylation is quite dynamic during cardiac stress. Further examination of the relationship between myofilament phosphorylation and cardiac diastolic dysfunction is required.

In conclusion, we have successfully developed a minipig HFpEF model characterized by cardiac fibrosis and remodeling. Our findings support the use of this porcine model of hypertension-induced HFpEF as a powerful tool to elucidate the mechanisms of this disease and translate preclinical findings.

## Limitations

Although this HFpEF model has multiple advantages, there are still some unsolved questions that require further investigation. For example, the molecular changes associated with this model were not fully investigated. Notably, stimulation with angiotensin II promotes cardiac fibroblast and collagen production (49). Exploring this signaling mechanism may help elucidate the pathogenesis of HFpEF in our pig model. Furthermore, consistency in the surgical procedure across laboratories may make generalization challenging. Additionally, our model only used one type of minipig. Because many laboratory pig strains have been used in different studies, it may be necessary to optimize the degree of constriction when using other strains to generate the model. Finally, Melleby et al. (17) have shown that a constriction ring induces HFpEF phenotypes, whereas a smaller ring leads to HFrEF. Despite these limitations, our study has established an effective model for basic or translational medical research and has uncovered crucial changes that provide mechanistic insights into HFpEF.

## Data Availability Statement

The datasets presented in this study can be found in online repositories. The names of the repository/repositories and accession number(s) can be found at: https://www.ncbi.nlm.nih.gov/geo/, GSE167643.

## Ethics Statement

The animal study was reviewed and approved by the Institutional Animal Care and Use Committee of the Guangdong Laboratory Animals Monitoring Institute (approval no. IACUC2017009).

## Author Contributions

FY, WT, and XianL designed and initiated the project. SZ, WT, XianL, XiaoL, FY, HS, XZ, JW, and HC were responsible for the laboratory experiments, data analysis, and/or animal care. WP, YZ, and PB provided critical comments during experiment design and manuscript preparation. All authors read and approved the final manuscript.

## Conflict of Interest

The authors declare that the research was conducted in the absence of any commercial or financial relationships that could be construed as a potential conflict of interest.

## References

[1] Maggioni AP Dahlström U Filippatos G Chioncel O Crespo Leiro M Drozdz J. EURObservational Research Programme: regional differences and 1-year follow-up results of the heart failure pilot survey (ESC-HF Pilot). Eur J Heart Fail. (2013) 15:808–17. 10.1093/eurjhf/hft050 23537547

[2] Virani SS Alonso A Benjamin EJ Bittencourt MS Callaway CW Carson AP. Heart disease and stroke statistics-2020 update: a report from the American Heart Association. Circulation. (2020) 141:e139–e596. 10.1161/CIR.0000000000000757 31992061

[3] Ma LY Chen WW Gao RL Liu LS Zhu ML Wang YJ. China cardiovascular diseases report 2018: an updated summary. J Geriatr Cardiol. (2020) 17:1–8. 10.11909/j.issn.1671-5411.2020.01.001 32133031 PMC7008101

[4] Ponikowski P Voors AA Anker SD Bueno H Cleland JGF Coats AJS. 2016 ESC Guidelines for the diagnosis treatment of acute chronic heart failure: the task force for the diagnosis treatment of acute chronic heart failure of the European Society of Cardiology (ESC) developed with the special contribution of the Heart Failure Association (HFA) of the ESC. Eur Heart J. (2016) 37:2129–200. 10.1093/eurheartj/ehw128 27206819

[5] Yancy CW Jessup M Bozkurt B Butler J Casey DE Jr Drazner MH. 2013 ACCF/AHA guideline for the management of heart failure: a report of the American College of Cardiology Foundation/American Heart Association Task Force on practice guidelines. Circulation. (2013) 128:e240–327. 10.1161/CIR.0b013e31829e8776 23741058

[6] Tsutsui H Isobe M Ito H Ito H Okumura K Ono M. JCS 2017/JHFS 2017 guideline on diagnosis and treatment of acute and chronic heart failure- digest version. Circ J. (2019) 83:2084–184. 10.1253/circj.CJ-19-0342 31511439

[7] Pfeffer MA Shah AM Borlaug BA. Heart failure with preserved ejection fraction in perspective. Circ Res. (2019) 124:1598–617. 10.1161/CIRCRESAHA.119.313572 31120821 PMC6534165

[8] Tromp J Teng TH Tay WT Hung CL Narasimhan C Shimizu W. Heart failure with preserved ejection fraction in Asia. Eur J Heart Fail. (2019) 21:23–36. 10.1002/ejhf.1227 30113120

[9] Cuijpers I Simmonds SJ Van Bilsen M Czarnowska E González Miqueo A Heymans S. Microvascular and lymphatic dysfunction in HFpEF and its associated comorbidities. Basic Res Cardiol. (2020) 115:39. 10.1007/s00395-020-0798-y 32451732 PMC7248044

[10] Mishra S Kass DA. Cellular and molecular pathobiology of heart failure with preserved ejection fraction. Nat Rev Cardiol. (2021). 10.1038/s41569-021-00516-5. [Epub ahead of print]. 33432192 PMC8574228

[11] Dixon JA Spinale FG. Large animal models of heart failure: a critical link in the translation of basic science to clinical practice. Circ Heart Fail. (2009) 2:262–71. 10.1161/CIRCHEARTFAILURE.108.814459 19808348 PMC2762217

[12] Riehle C Bauersachs J. Small animal models of heart failure. Cardiovasc Res. (2019) 115:1838–49. 10.1093/cvr/cvz161 31243437 PMC6803815

[13] Schunkert H Dzau VJ Tang SS Hirsch AT Apstein CS Lorell BH. Increased rat cardiac angiotensin converting enzyme activity and mRNA expression in pressure overload left ventricular hypertrophy. Effects on coronary resistance, contractility, and relaxation. J Clin Invest. (1990) 86:1913–20. 10.1172/JCI114924 2174912 PMC329826

[14] Rockman HA Ross RS Harris AN Knowlton KU Steinhelper ME Field LJ. Segregation of atrial-specific and inducible expression of an atrial natriuretic factor transgene in an *in vivo* murine model of cardiac hypertrophy. Proc Natl Acad Sci U.S.A. (1991) 88:8277–81. 10.1073/pnas.88.18.8277 1832775 PMC52490

[15] Weinberg EO Schoen FJ George D Kagaya Y Douglas PS Litwin SE. Angiotensin-converting enzyme inhibition prolongs survival and modifies the transition to heart failure in rats with pressure overload hypertrophy due to ascending aortic stenosis. Circulation. (1994) 90:1410–22. 10.1161/01.CIR.90.3.1410 8087951

[16] Litwin SE Katz SE Weinberg EO Lorell BH Aurigemma GP Douglas PS. Serial echocardiographic-Doppler assessment of left ventricular geometry and function in rats with pressure-overload hypertrophy. Chronic angiotensin-converting enzyme inhibition attenuates the transition to heart failure. Circulation. (1995) 91:2642–54. 10.1161/01.CIR.91.10.2642 7743628

[17] Melleby AO Romaine A Aronsen JM Veras I Zhang L Sjaastad I. A novel method for high precision aortic constriction that allows for generation of specific cardiac phenotypes in mice. Cardiovasc Res. (2018) 114:1680–90. 10.1093/cvr/cvy141 29878127

[18] Charles CJ Lee P Li RR Yeung T Ibraham Mazlan SM Tay ZW. A porcine model of heart failure with preserved ejection fraction: magnetic resonance imaging and metabolic energetics. ESC Heart Fail. (2020) 7:92–102. 10.1002/ehf2.12536 31851785 PMC7083424

[19] Li X Zheng S Tan W Chen H Li X Wu J. Slit2 protects hearts against ischemia-reperfusion injury by inhibiting inflammatory responses and maintaining myofilament contractile properties. Front Physiol. (2020) 11:228. 10.3389/fphys.2020.00228 32292352 PMC7135862

[20] Yang FH Pyle WG. Reduced cardiac CapZ protein protects hearts against acute ischemia-reperfusion injury and enhances preconditioning. J Mol Cell Cardiol. (2012) 52:761–72. 10.1016/j.yjmcc.2011.11.013 22155006

[21] Zheng S Tan W Li X Li B Gong B Pyle WG. Aged monkeys fed a high-fat/high-sugar diet recapitulate metabolic disorders and cardiac contractile dysfunction. J Cardiovasc Transl Res. (2021). 10.1007/s12265-021-10105-z. [Epub ahead of print]. 33591467

[22] Schwarzl M Hamdani N Seiler S Alogna A Manninger M Reilly S. A porcine model of hypertensive cardiomyopathy: implications for heart failure with preserved ejection fraction. Am J Physiol Heart Circ Physiol. (2015) 309:H1407–18. 10.1152/ajpheart.00542.2015 26342070

[23] Hamdani N Bishu KG Von Frieling-Salewsky M Redfield MM Linke WA. Deranged myofilament phosphorylation and function in experimental heart failure with preserved ejection fraction. Cardiovasc Res. (2013) 97:464–71. 10.1093/cvr/cvs353 23213108

[24] Shah AM Pfeffer MA. The many faces of heart failure with preserved ejection fraction. Nat Rev Cardiol. (2012) 9:555–6. 10.1038/nrcardio.2012.123 22945329

[25] Glezeva N Baugh JA. Role of inflammation in the pathogenesis of heart failure with preserved ejection fraction and its potential as a therapeutic target. Heart Fail Rev. (2014) 19:681–94. 10.1007/s10741-013-9405-8 24005868

[26] Gyöngyösi M Pavo N Lukovic D Zlabinger K Spannbauer A Traxler D. Porcine model of progressive cardiac hypertrophy and fibrosis with secondary postcapillary pulmonary hypertension. J Transl Med. (2017) 15:202. 10.1186/s12967-017-1299-0 28985746 PMC5639584

[27] Bikou O Miyashita S Ishikawa K. Pig model of increased cardiac afterload induced by ascending aortic banding. Methods Mol Biol. (2018) 1816:337–42. 10.1007/978-1-4939-8597-5_26 29987832

[28] Hiemstra JA Veteto AB Lambert MD Olver TD Ferguson BS Mcdonald KS. Chronic low-intensity exercise attenuates cardiomyocyte contractile dysfunction and impaired adrenergic responsiveness in aortic-banded mini-swine. J Appl Physiol. (1985) 124:1034–44. 10.1152/japplphysiol.00840.2017 29357490 PMC5972453

[29] Olver TD Edwards JC Ferguson BS Hiemstra JA Thorne PK Hill MA. Chronic interval exercise training prevents BK(Ca) channel-mediated coronary vascular dysfunction in aortic-banded miniswine. J Appl Physiol. (2018) 125:86–96. 29596016 10.1152/japplphysiol.01138.2017 PMC6086974

[30] Reiter U Reiter G Manninger M Adelsmayr G Schipke J Alogna A. Early-stage heart failure with preserved ejection fraction in the pig: a cardiovascular magnetic resonance study. J Cardiovasc Magn Reson. (2016) 18:63. 10.1186/s12968-016-0283-9 27688028 PMC5043627

[31] Silva KS Leary EV Olver TD Domeier TL Padilla J Rector RS. Tissue-specific small heat shock protein 20 activation is not associated with traditional autophagy markers in Ossabaw swine with cardiometabolic heart failure. Am J Physiol Heart Circ Physiol. (2020) 319:H1036–43. 10.1152/ajpheart.00580.2020 32946285 PMC7789972

[32] Deng Y Xie M Li Q Xu X Ou W Zhang Y. Targeting mitochondria-inflammation circuit by β-hydroxybutyrate mitigates HFpEF. Circ Res. (2021) 128:232–45. 10.1161/CIRCRESAHA.120.317933 33176578

[33] Hage C Löfgren L Michopoulos F Nilsson R Davidsson P Kumar C. Metabolomic profile in HFpEF vs HFrEF patients. J Card Fail. (2020) 26:1050–9. 10.1016/j.cardfail.2020.07.010 32750486

[34] Khalil H Kanisicak O Prasad V Correll RN Fu X Schips T. Fibroblast-specific TGF-β-Smad2/3 signaling underlies cardiac fibrosis. J Clin Invest. (2017) 127:3770–83. 10.1172/JCI94753 28891814 PMC5617658

[35] Leslie KO Taatjes DJ Schwarz J Vonturkovich M Low RB. Cardiac myofibroblasts express alpha smooth muscle actin during right ventricular pressure overload in the rabbit. Am J Pathol. (1991) 139:207–16. 1853934 PMC1886148

[36] Phosri S Arieyawong A Bunrukchai K Parichatikanond W Nishimura A Nishida M. Stimulation of adenosine A(2B) receptor inhibits endothelin-1-induced cardiac fibroblast proliferation and α-smooth muscle actin synthesis through the cAMP/Epac/PI3K/Akt-signaling pathway. Front Pharmacol. (2017) 8:428. 10.3389/fphar.2017.00428 28713274 PMC5492828

[37] Sun S Kee HJ Jin L Ryu Y Choi SY Kim GR. Gentisic acid attenuates pressure overload-induced cardiac hypertrophy and fibrosis in mice through inhibition of the ERK1/2 pathway. J Cell Mol Med. (2018) 22:5964–77. 10.1111/jcmm.13869 30256522 PMC6237595

[38] Bergman MR Teerlink JR Mahimkar R Li L Zhu BQ Nguyen A. Cardiac matrix metalloproteinase-2 expression independently induces marked ventricular remodeling and systolic dysfunction. Am J Physiol Heart Circ Physiol. (2007) 292:H1847–60. 10.1152/ajpheart.00434.2006 17158653

[39] Heymans S Lupu F Terclavers S Vanwetswinkel B Herbert JM Baker A. Loss or inhibition of uPA or MMP-9 attenuates LV remodeling and dysfunction after acute pressure overload in mice. Am J Pathol. (2005) 166:15–25. 10.1016/S0002-9440(10)62228-6 15631996 PMC1602291

[40] Lindsay MM Maxwell P Dunn FG. TIMP-1: a marker of left ventricular diastolic dysfunction and fibrosis in hypertension. Hypertension. (2002) 40:136–41. 10.1161/01.HYP.0000024573.17293.23 12154103

[41] Kandalam V Basu R Moore L Fan D Wang X Jaworski DM. Lack of tissue inhibitor of metalloproteinases 2 leads to exacerbated left ventricular dysfunction and adverse extracellular matrix remodeling in response to biomechanical stress. Circulation. (2011) 124:2094–105. 10.1161/CIRCULATIONAHA.111.030338 21986284

[42] Polyakova V Loeffler I Hein S Miyagawa S Piotrowska I Dammer S. Fibrosis in endstage human heart failure: severe changes in collagen metabolism and MMP/TIMP profiles. Int J Cardiol. (2011) 151:18–433. 10.1016/j.ijcard.2010.04.053 20546954

[43] Borlaug BA. The pathophysiology of heart failure with preserved ejection fraction. Nat Rev Cardiol. (2014) 11:507–15. 10.1038/nrcardio.2014.83 24958077

[44] Obokata M Reddy YNV Borlaug BA. Diastolic dysfunction and heart failure with preserved ejection fraction: understanding mechanisms by using noninvasive methods. JACC Cardiovasc Imaging. (2020) 13:245–57. 10.1016/j.jcmg.2018.12.034 31202759 PMC6899218

[45] Bacmeister L Schwarzl M Warnke S Stoffers B Blankenberg S Westermann D. Inflammation and fibrosis in murine models of heart failure. Basic Res Cardiol. (2019) 114:19. 10.1007/s00395-019-0722-5 30887214

[46] Kalogeropoulos A Georgiopoulou V Psaty BM Rodondi N Smith AL Harrison DG. Inflammatory markers and incident heart failure risk in older adults: the health ABC (health, aging, and body composition) study. J Am Coll Cardiol. (2010) 55:2129–37. 10.1016/j.jacc.2009.12.045 20447537 PMC3267799

[47] Kuwahara F Kai H Tokuda K Takeya M Takeshita A Egashira K. Hypertensive myocardial fibrosis and diastolic dysfunction: another model of inflammation? Hypertension. (2004) 43:739–45. 10.1161/01.HYP.0000118584.33350.7d 14967845

[48] Rosas PC Liu Y Abdalla MI Thomas CM Kidwell DT Dusio GF. Phosphorylation of cardiac Myosin-binding protein-C is a critical mediator of diastolic function. Circ Heart Fail. (2015) 8:582–94. 10.1161/CIRCHEARTFAILURE.114.001550 25740839 PMC4447128

[49] Sadoshima J Izumo S. Molecular characterization of angiotensin II–induced hypertrophy of cardiac myocytes and hyperplasia of cardiac fibroblasts. Critical role of the AT1 receptor subtype. Circ Res. (1993) 73:413–23. 10.1161/01.RES.73.3.413 8348686

