# Supplementary material for: A Porcine Model of Heart Failure With Preserved Ejection Fraction Induced by Chronic Pressure Overload Characterized by Cardiac Fibrosis and Remodeling
Source: Front Cardiovasc Med. 2021 Jun 2;8:677727. doi: 10.3389/fcvm.2021.677727 (PMC8206269; doi:10.3389/fcvm.2021.677727)

col1a1 ⇒

GAPDH ⇒

p-ERK ⇒

GAPDH ⇒

p-PI3K ⇒

GAPDH ⇒

IL-6 ⇒

(GAPDH 同 p-PI3K GAPDH)

p-NFKB ⇒

GAPDH ⇒

p-Smad2/3 ⇒

GAPDH ⇒

p-Smad2 } TGF-β } ⇒

GAPDH同 p-IKBα GAPDH)

p-IKBα } GAPDH } ⇒

IL-1β } GAPDH } ⇒

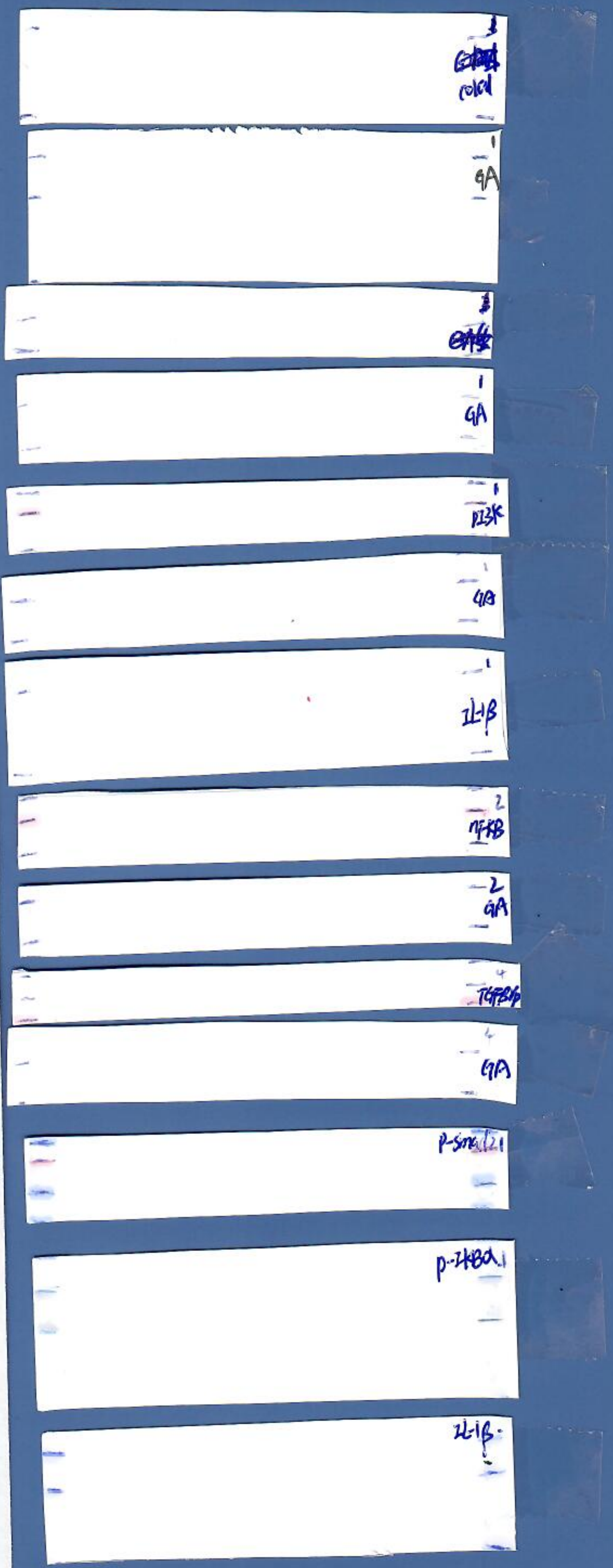

Supplement: Supplementary file 1 [file Data_Sheet_1.ZIP › Membranes for WB/Membranes for WB.pdf]
