# Supplementary figures and images for: A Porcine Model of Heart Failure With Preserved Ejection Fraction Induced by Chronic Pressure Overload Characterized by Cardiac Fibrosis and Remodeling
Source: Front Cardiovasc Med. 2021 Jun 2;8:677727. doi: 10.3389/fcvm.2021.677727 (PMC8206269; doi:10.3389/fcvm.2021.677727)

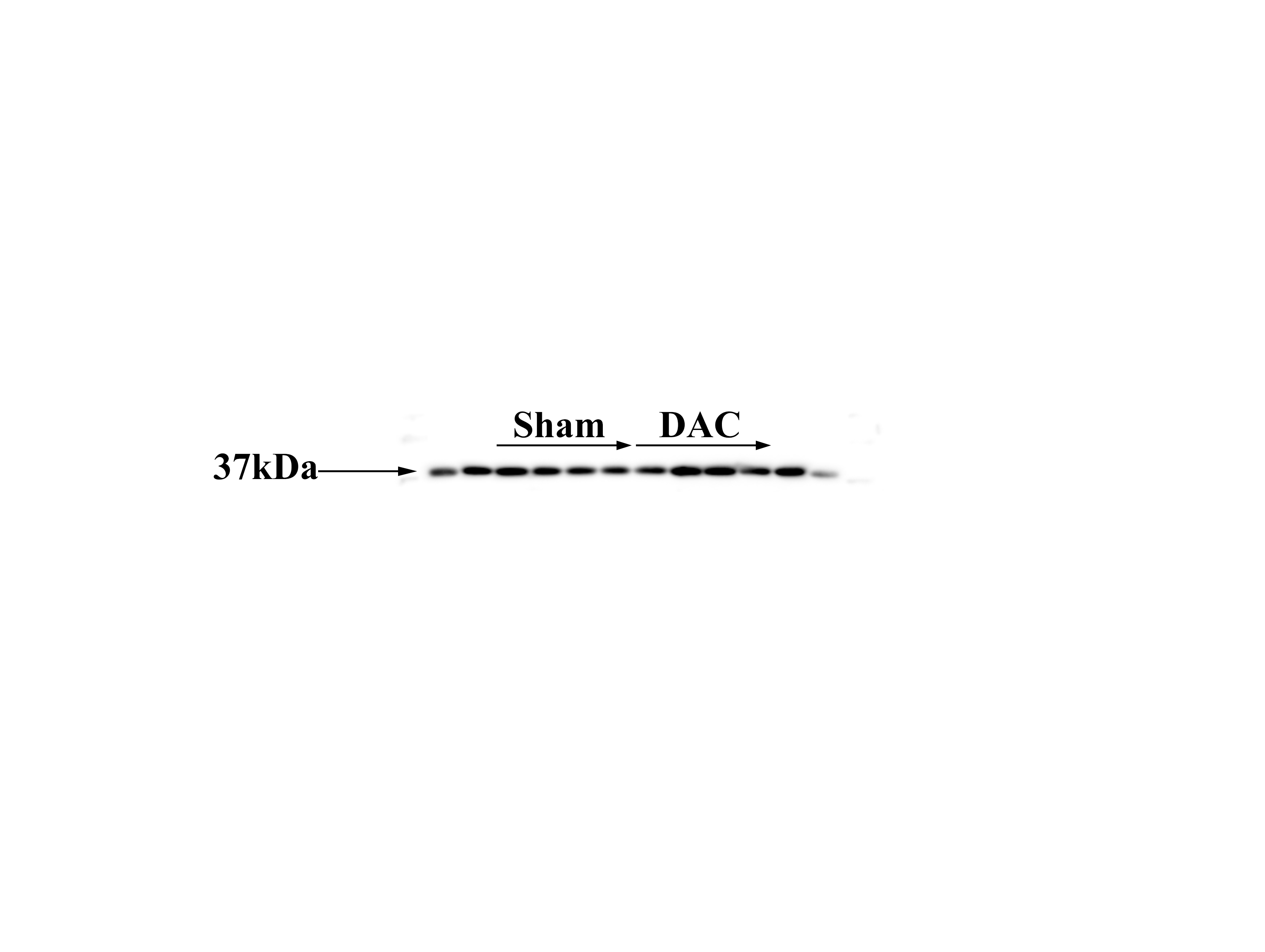

Supplement: Supplementary file 1 [file Data_Sheet_1.ZIP › Membranes for WB/1 GAPDH for IL-6.tif]

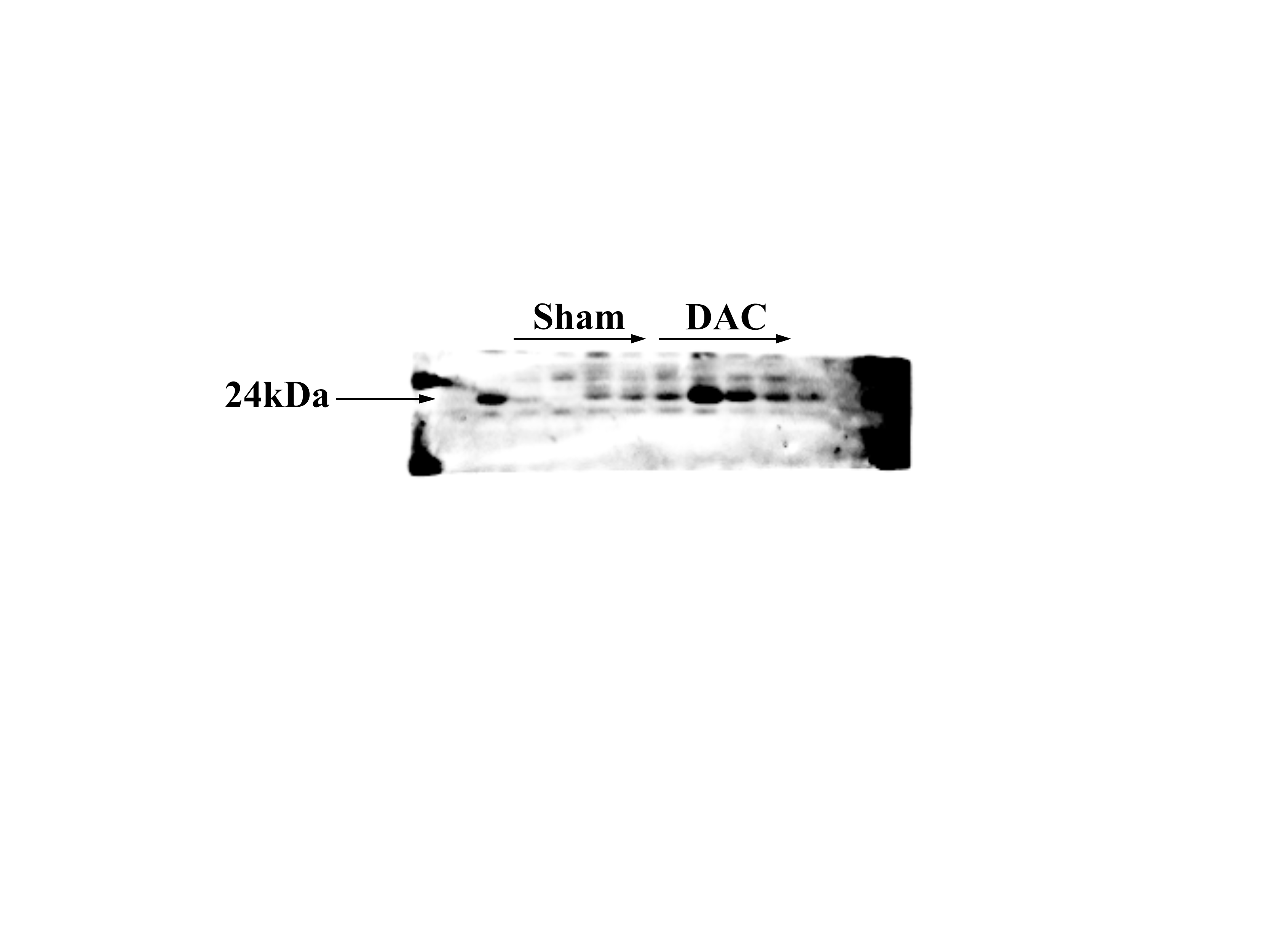

Supplement: Supplementary file 1 [file Data_Sheet_1.ZIP › Membranes for WB/1 IL-6.tif]

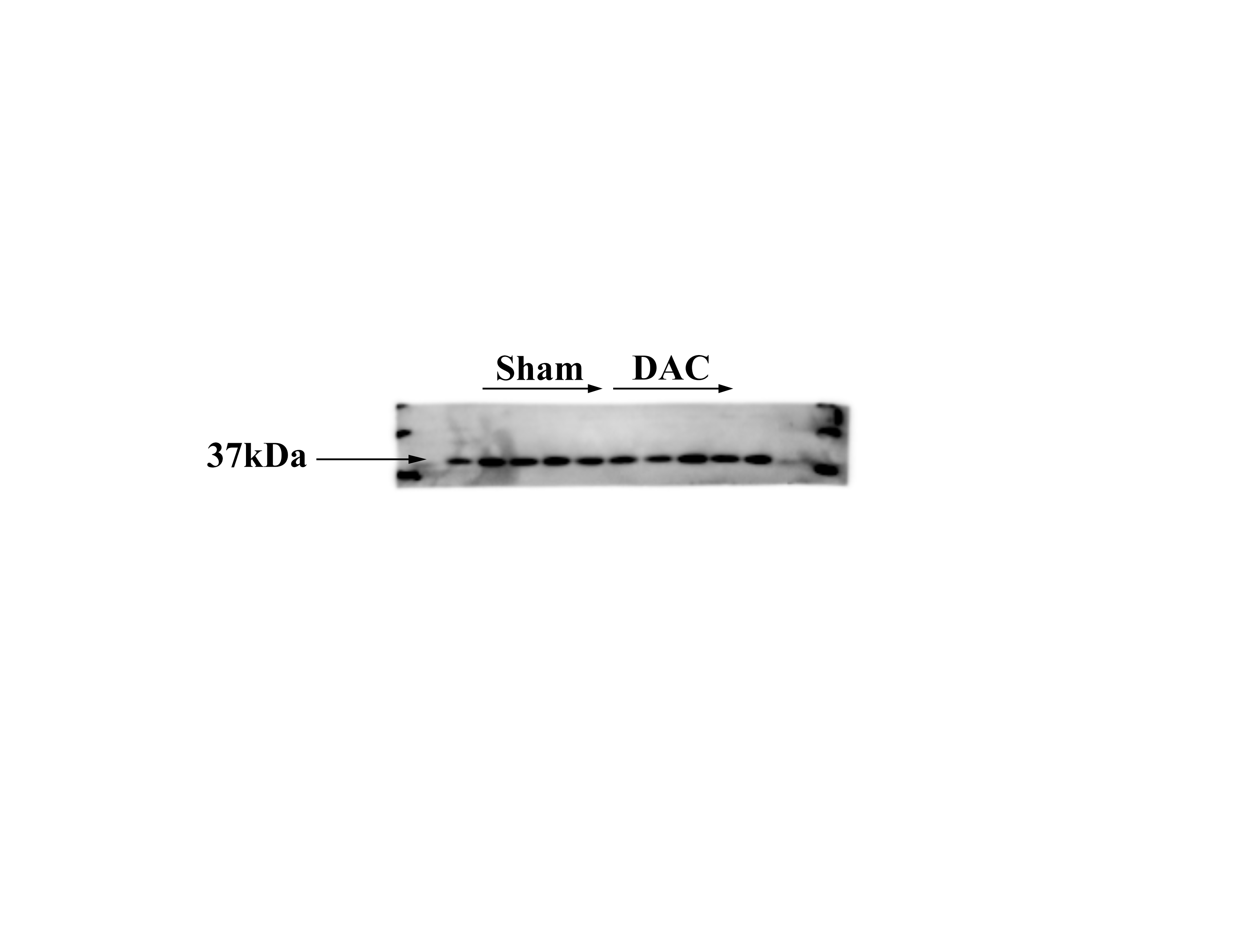

Supplement: Supplementary file 1 [file Data_Sheet_1.ZIP › Membranes for WB/10 GAPDH for p-ERK.tif]

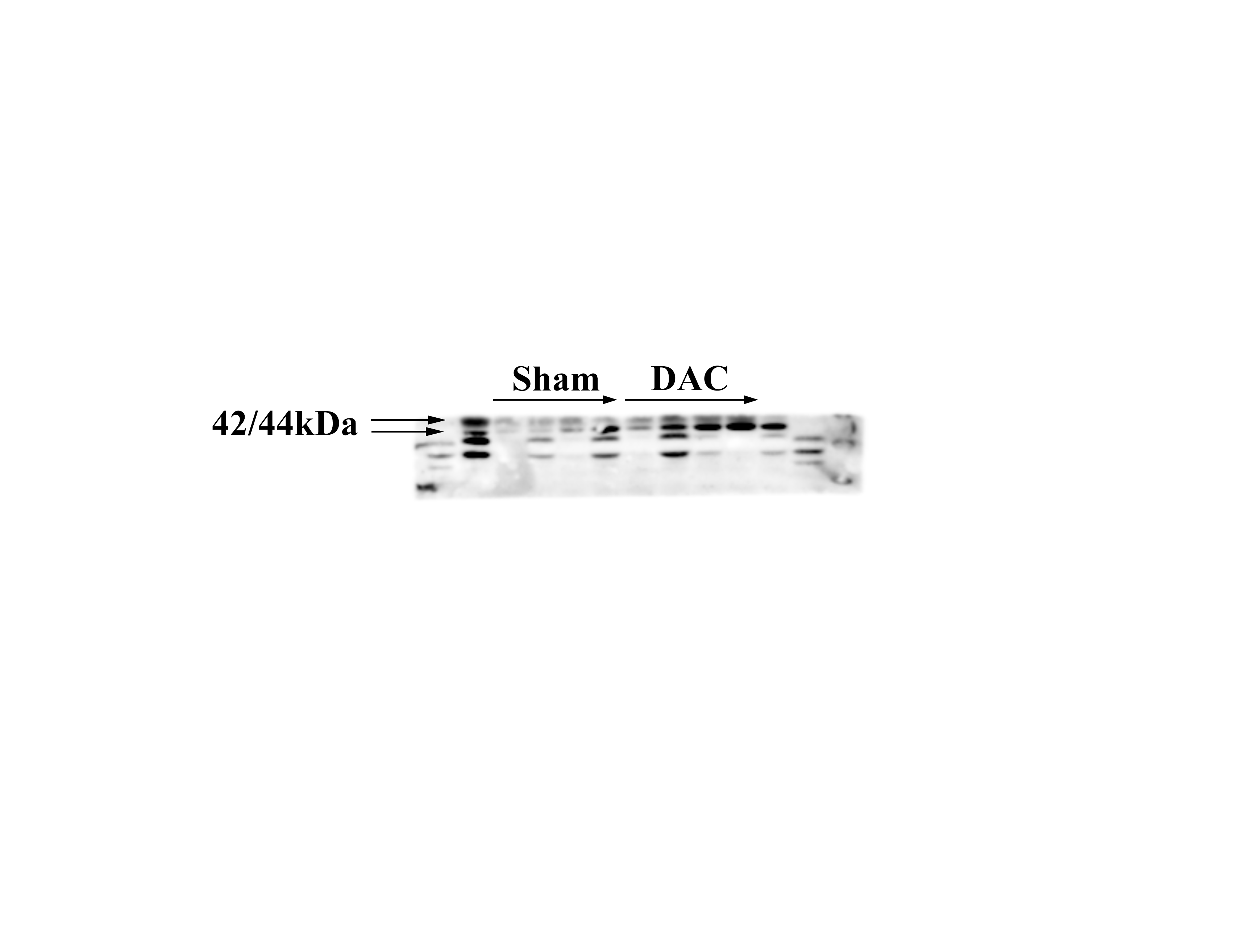

Supplement: Supplementary file 1 [file Data_Sheet_1.ZIP › Membranes for WB/10 p-ERK.tif]

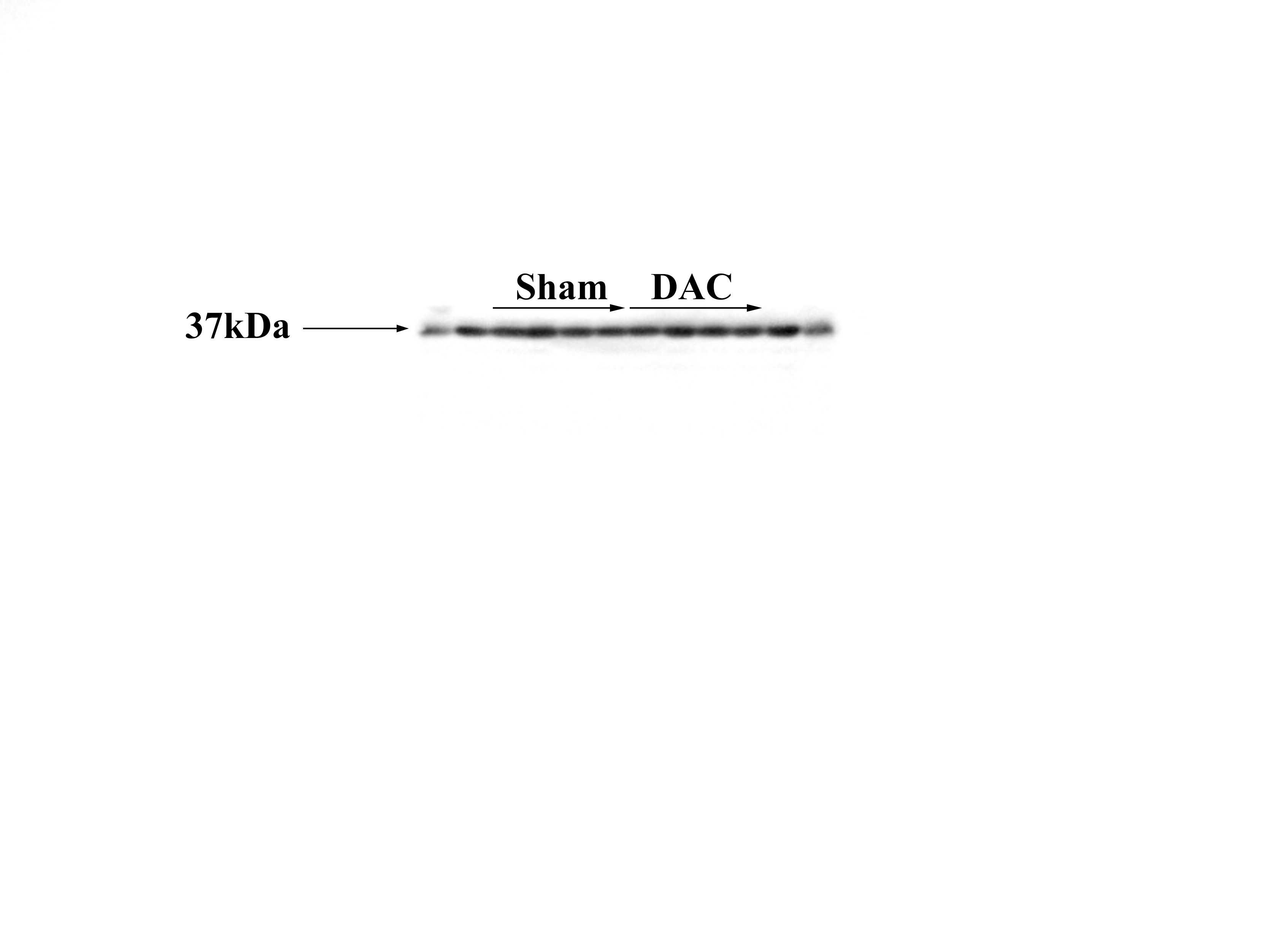

Supplement: Supplementary file 1 [file Data_Sheet_1.ZIP › Membranes for WB/2 GAPDH for IL-1a┬.tif]

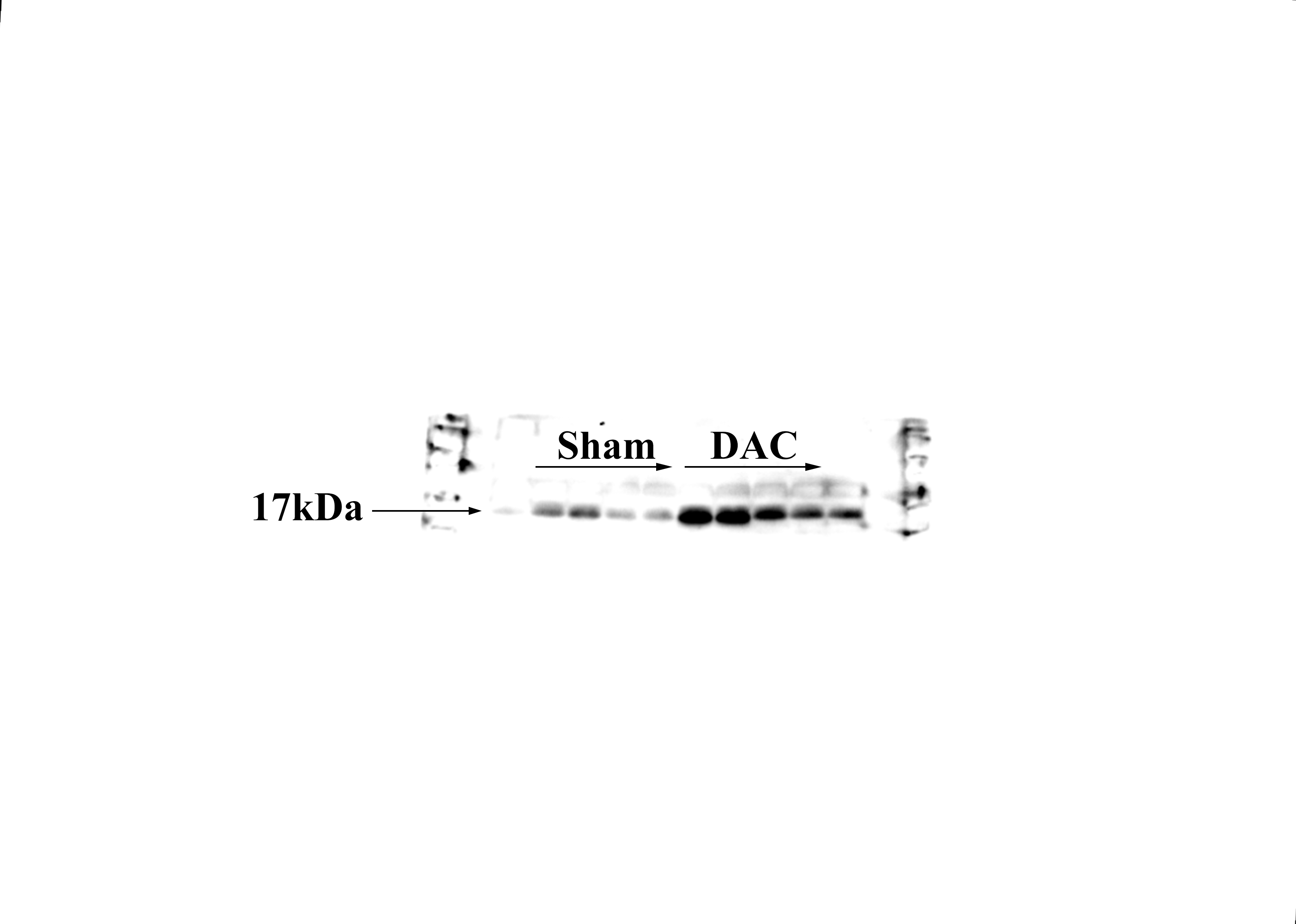

Supplement: Supplementary file 1 [file Data_Sheet_1.ZIP › Membranes for WB/2 IL-1a┬.tif]

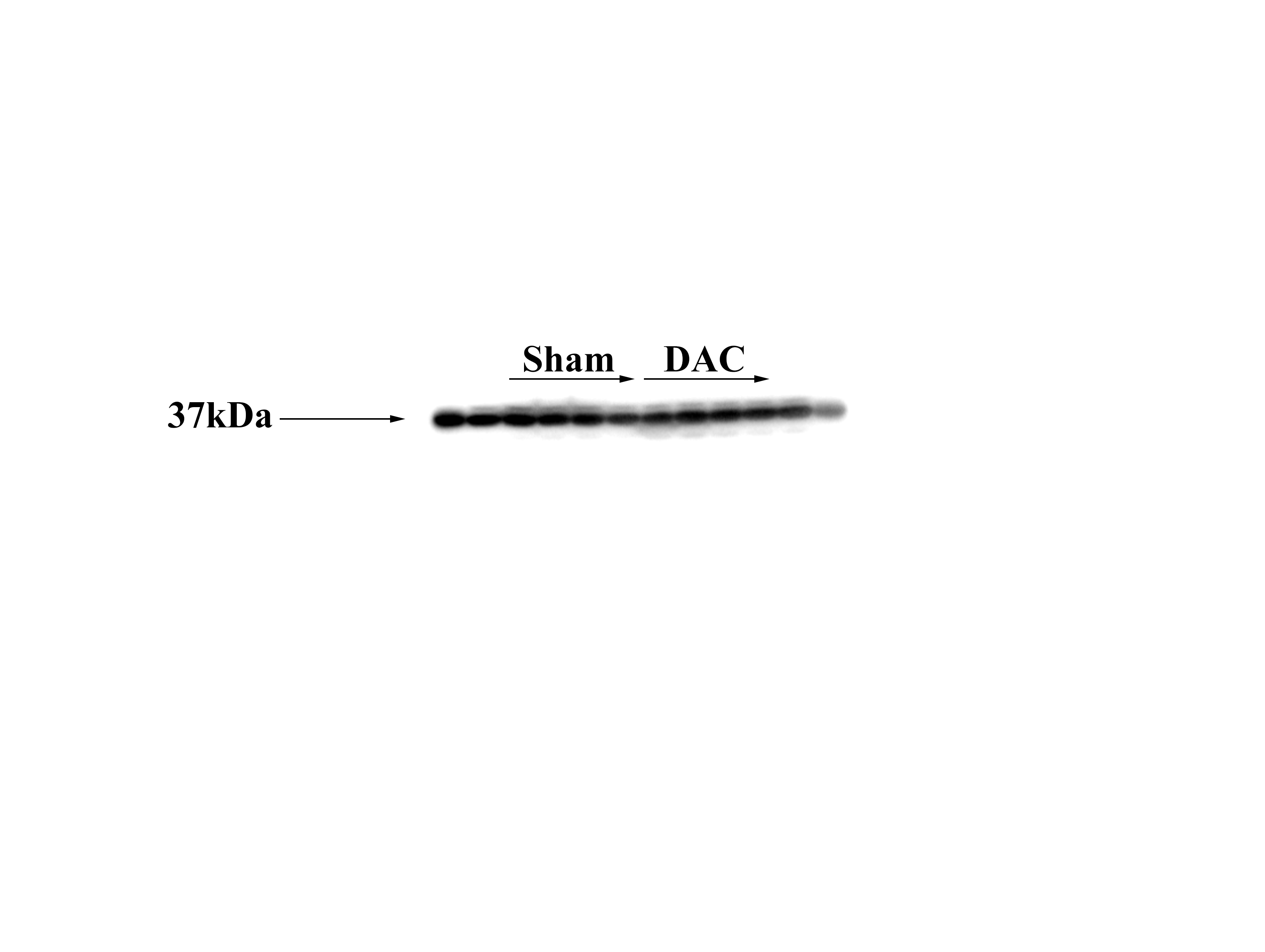

Supplement: Supplementary file 1 [file Data_Sheet_1.ZIP › Membranes for WB/3 GAPDH for p-IkappaBa┴.tif]

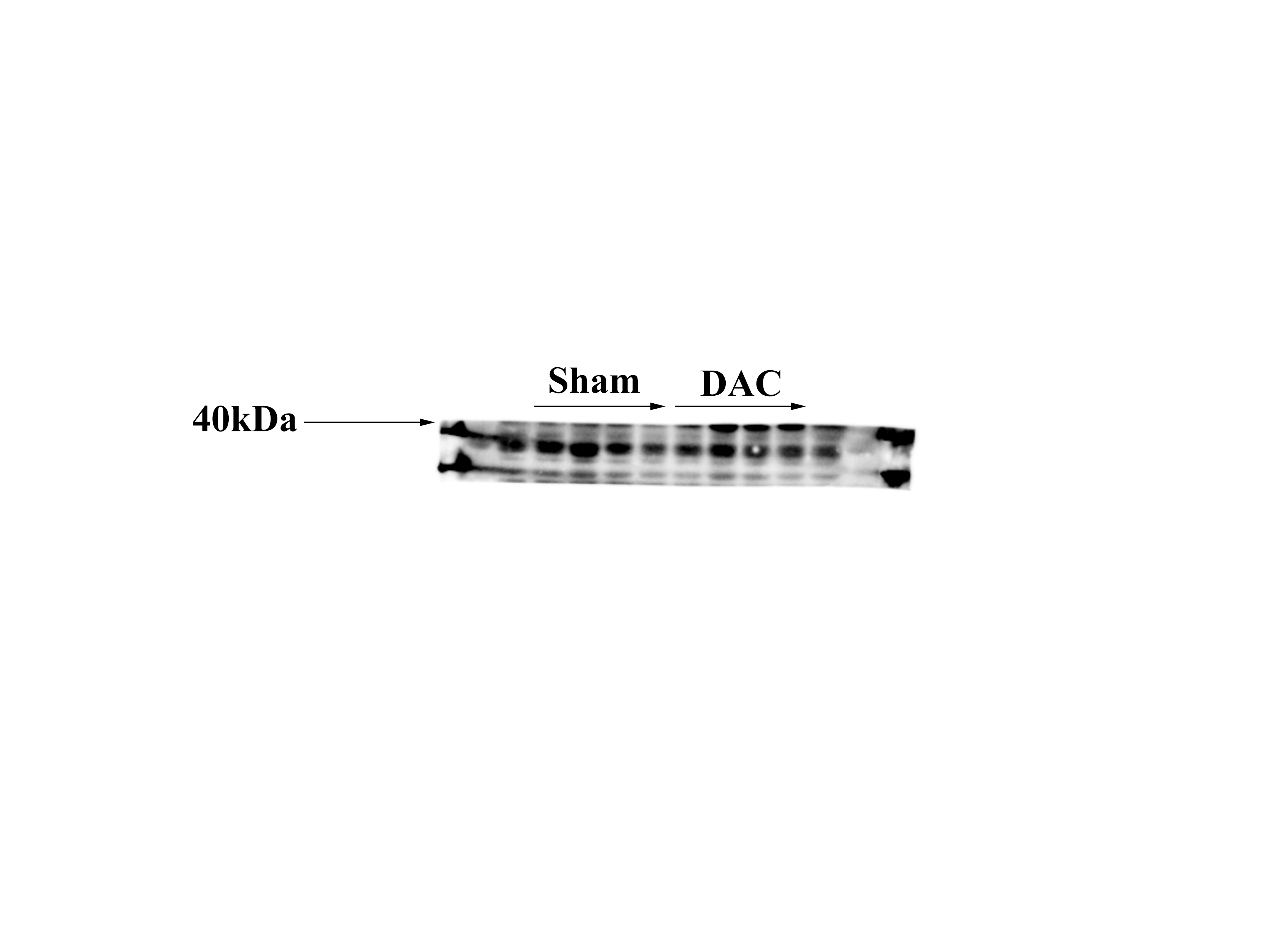

Supplement: Supplementary file 1 [file Data_Sheet_1.ZIP › Membranes for WB/3 p-IkappaBa┴.tif]

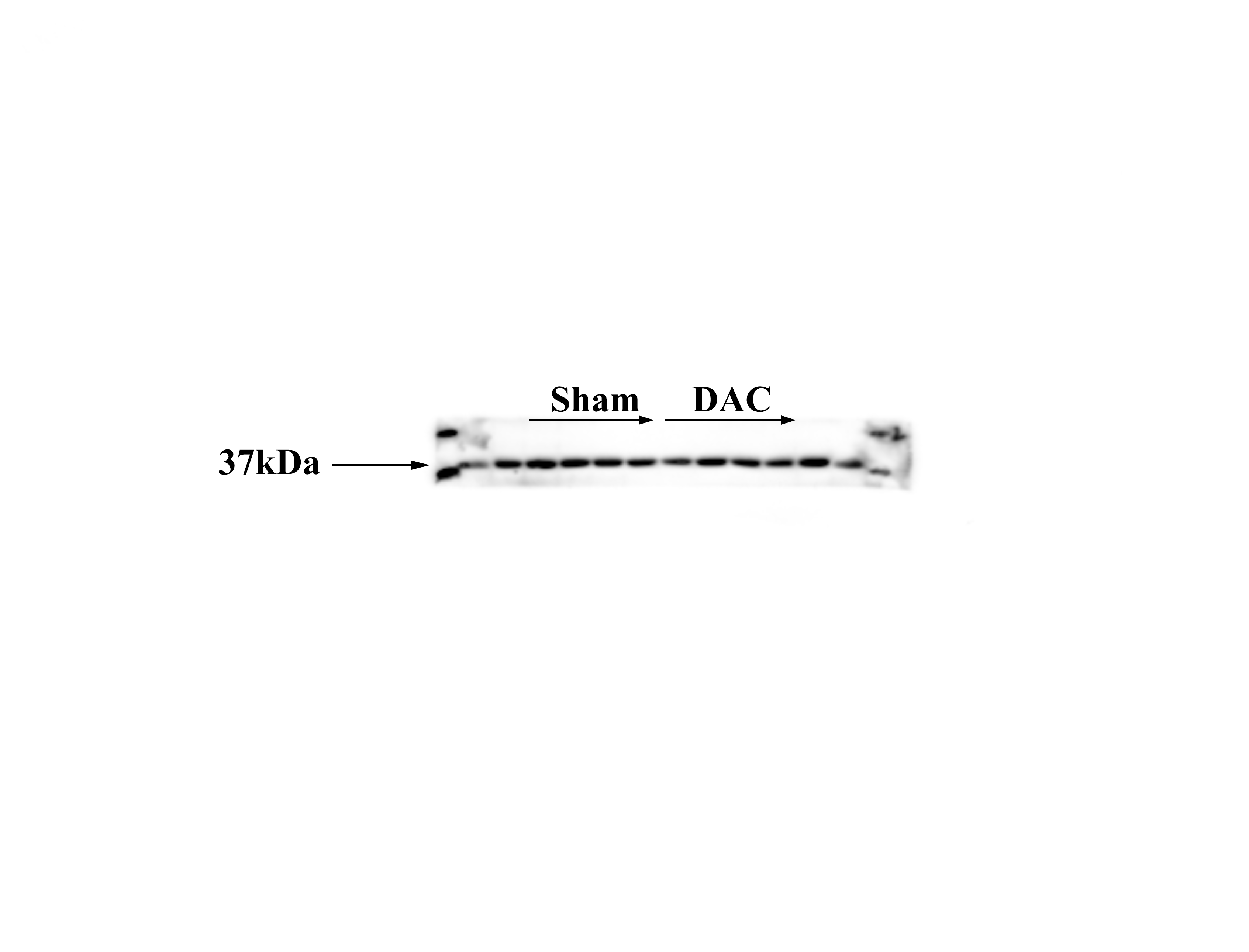

Supplement: Supplementary file 1 [file Data_Sheet_1.ZIP › Membranes for WB/4 GAPDH for p-NFkappaB.tif]

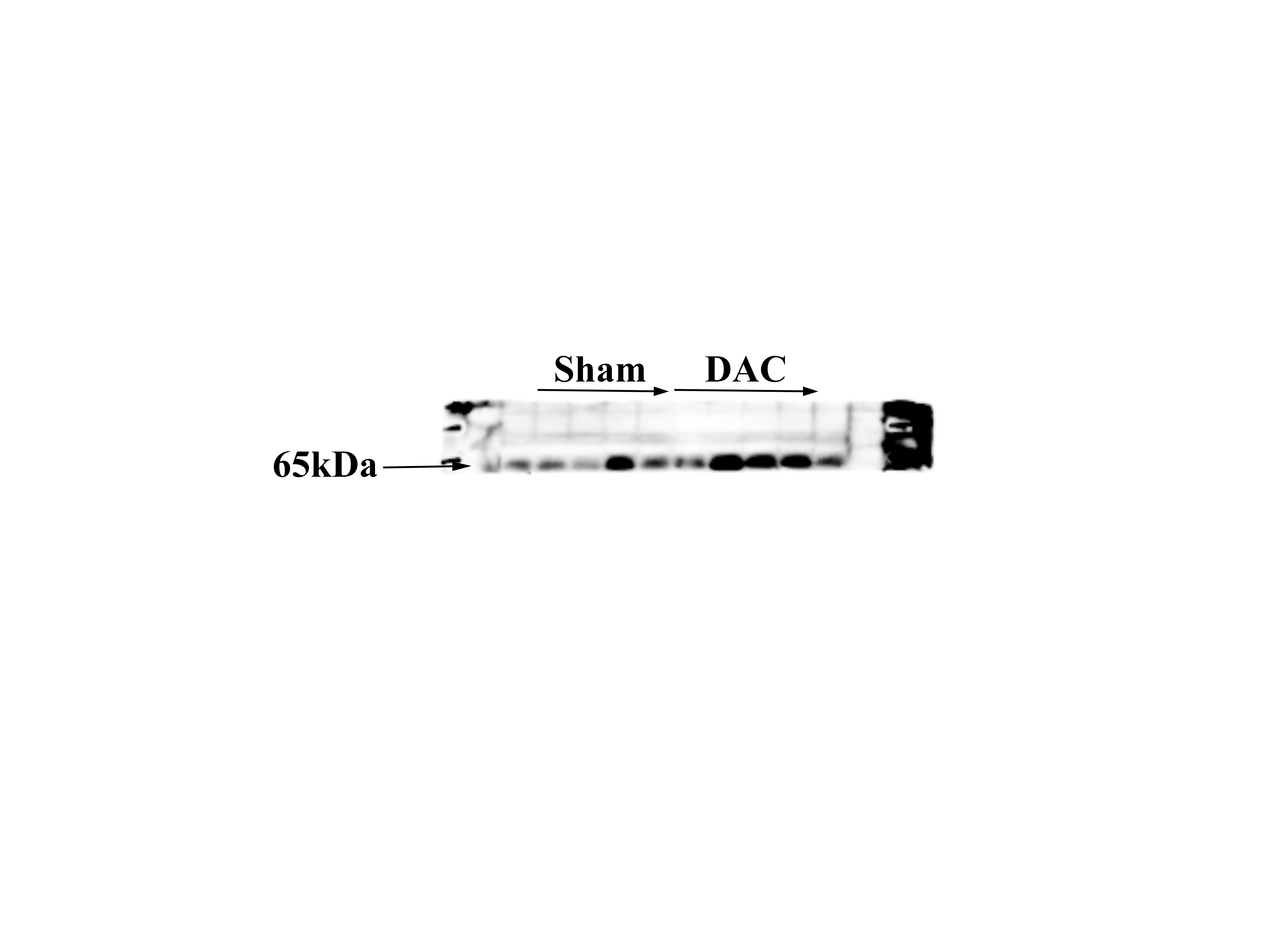

Supplement: Supplementary file 1 [file Data_Sheet_1.ZIP › Membranes for WB/4 p-NFkappaB.tif]

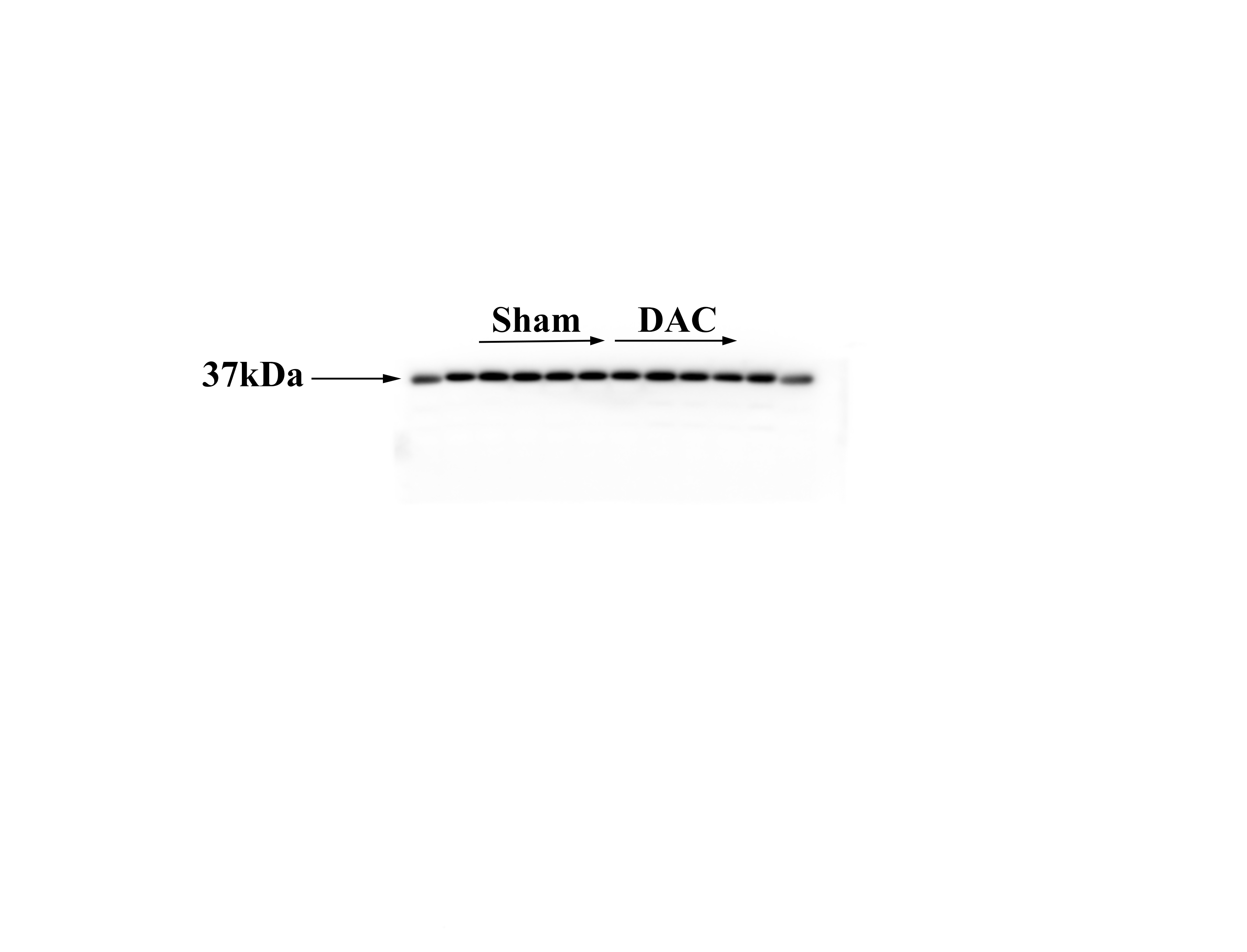

Supplement: Supplementary file 1 [file Data_Sheet_1.ZIP › Membranes for WB/5 GAPGH for col1a┴1.tif]

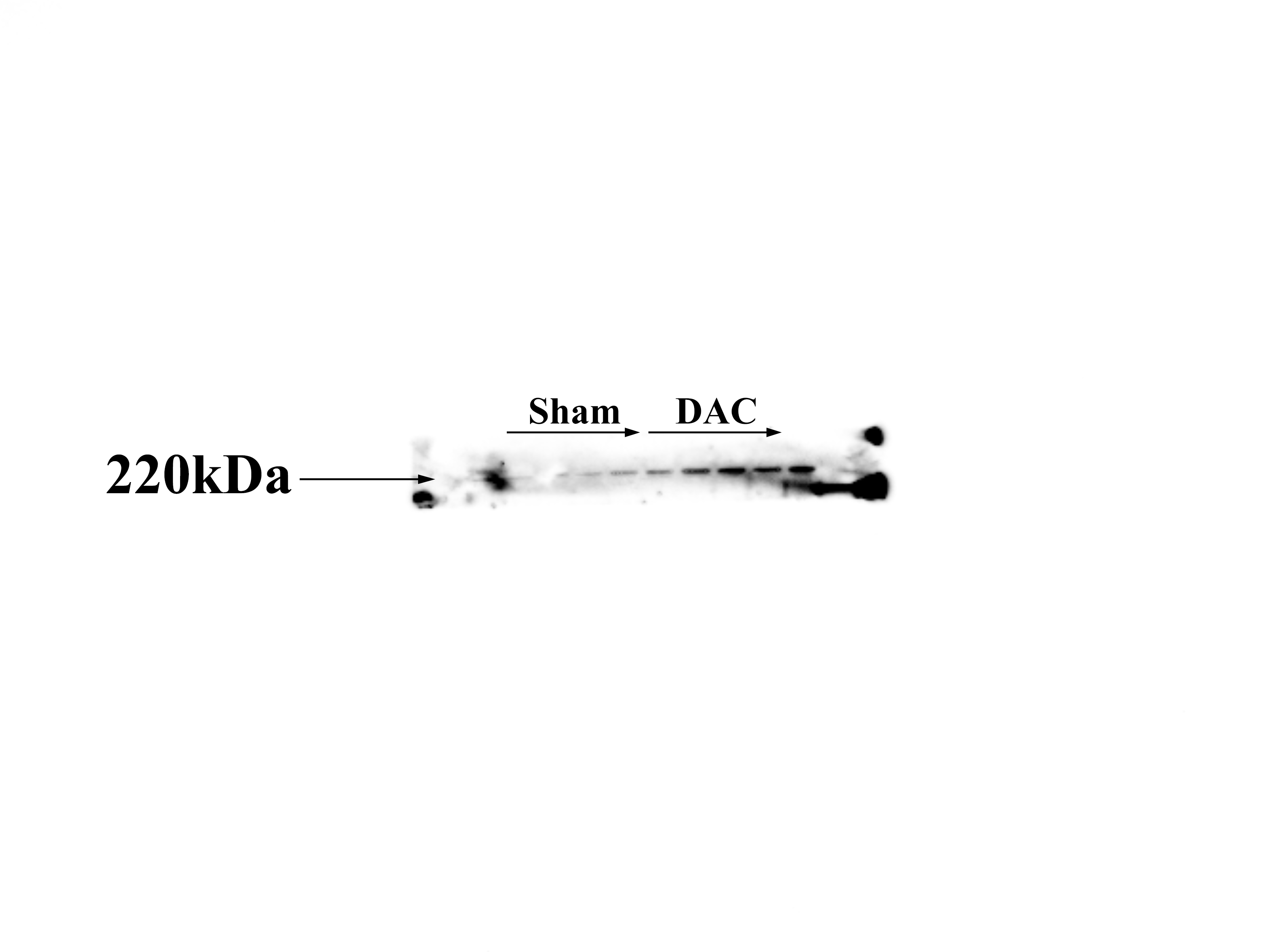

Supplement: Supplementary file 1 [file Data_Sheet_1.ZIP › Membranes for WB/5 col1a┴1.tif]

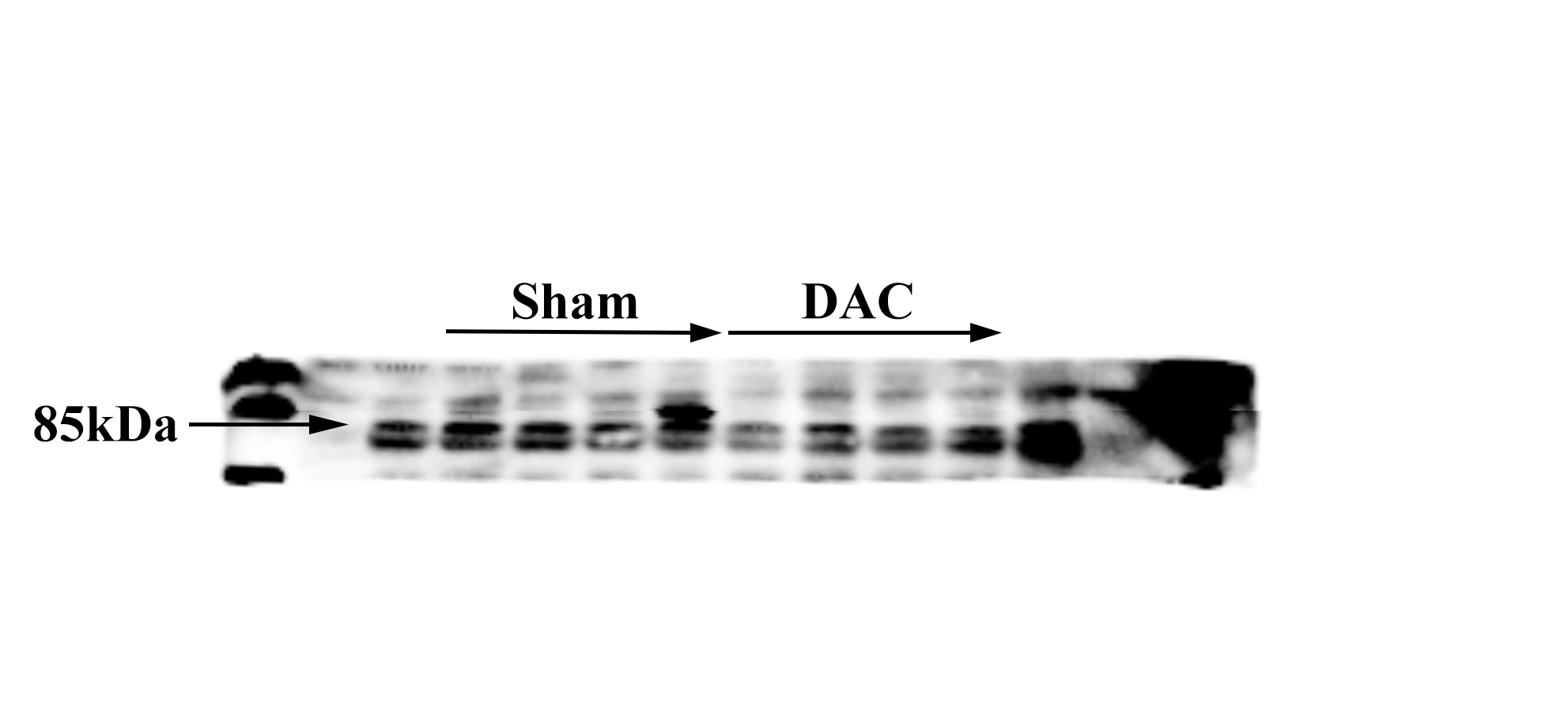

Supplement: Supplementary file 1 [file Data_Sheet_1.ZIP › Membranes for WB/9 p-PI3K.tif]

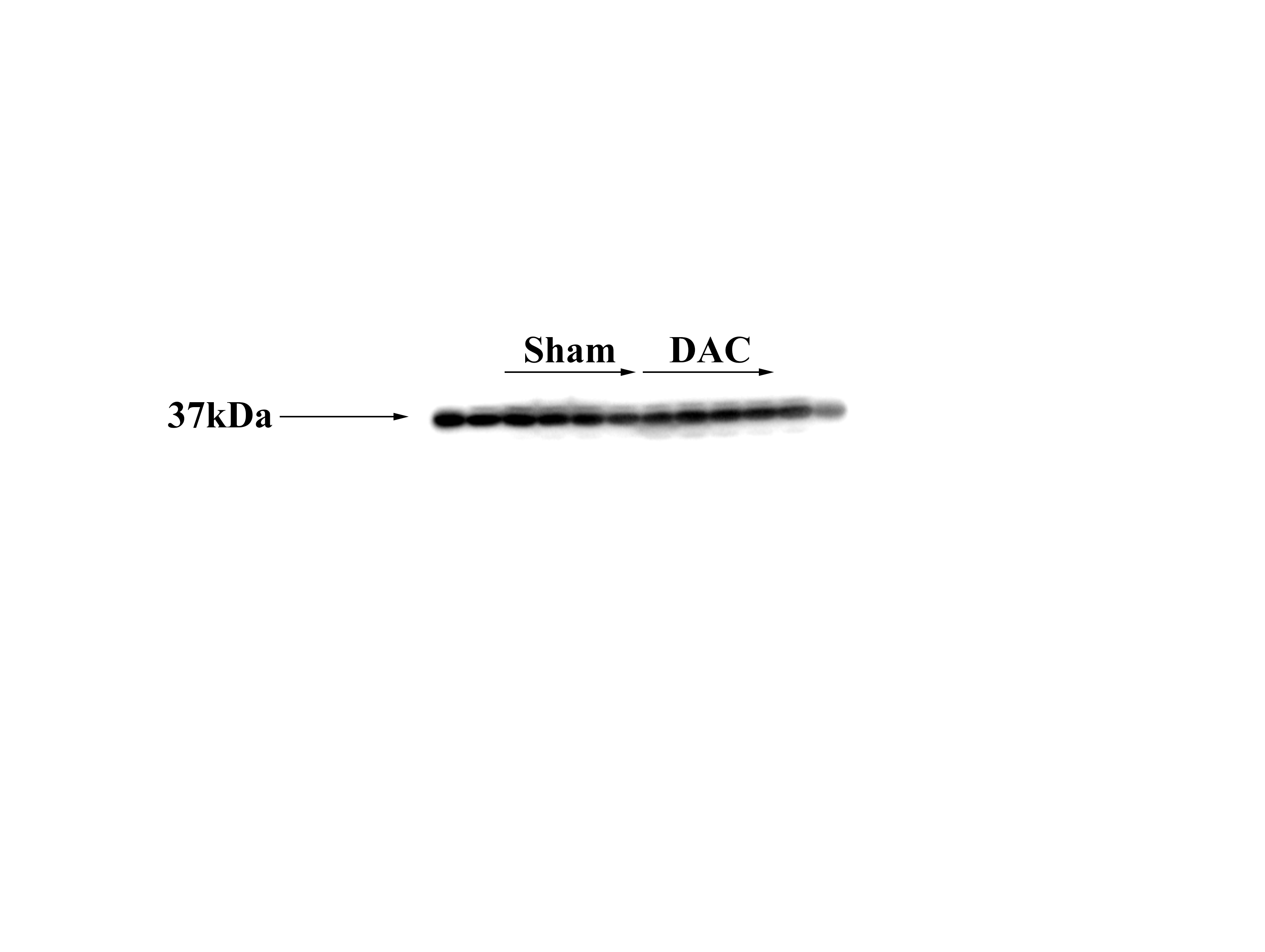

Supplement: Supplementary file 1 [file Data_Sheet_1.ZIP › Membranes for WB/6 GAPDH for TGF-a┬.tif]

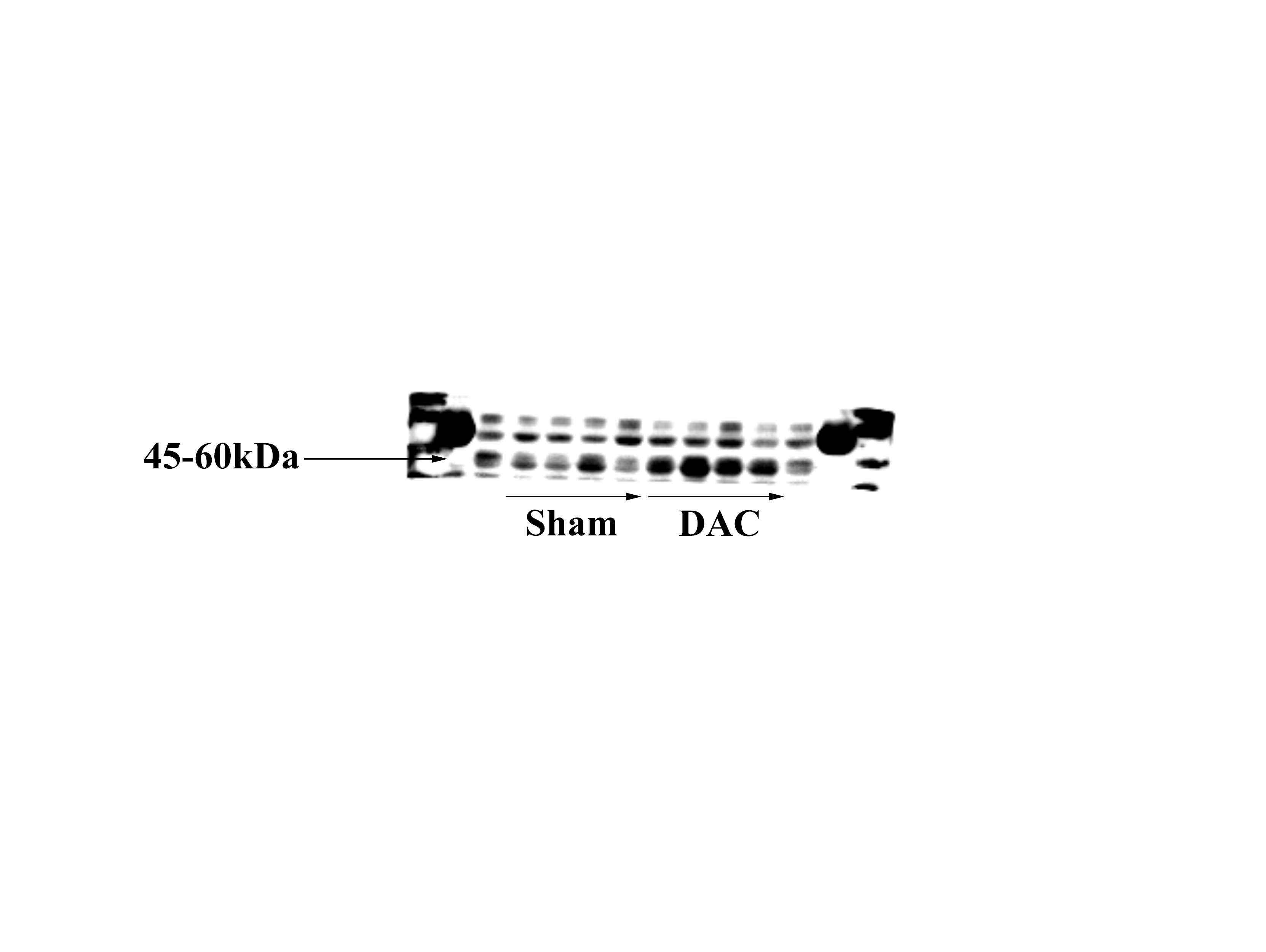

Supplement: Supplementary file 1 [file Data_Sheet_1.ZIP › Membranes for WB/6 TGF-a┬.tif]

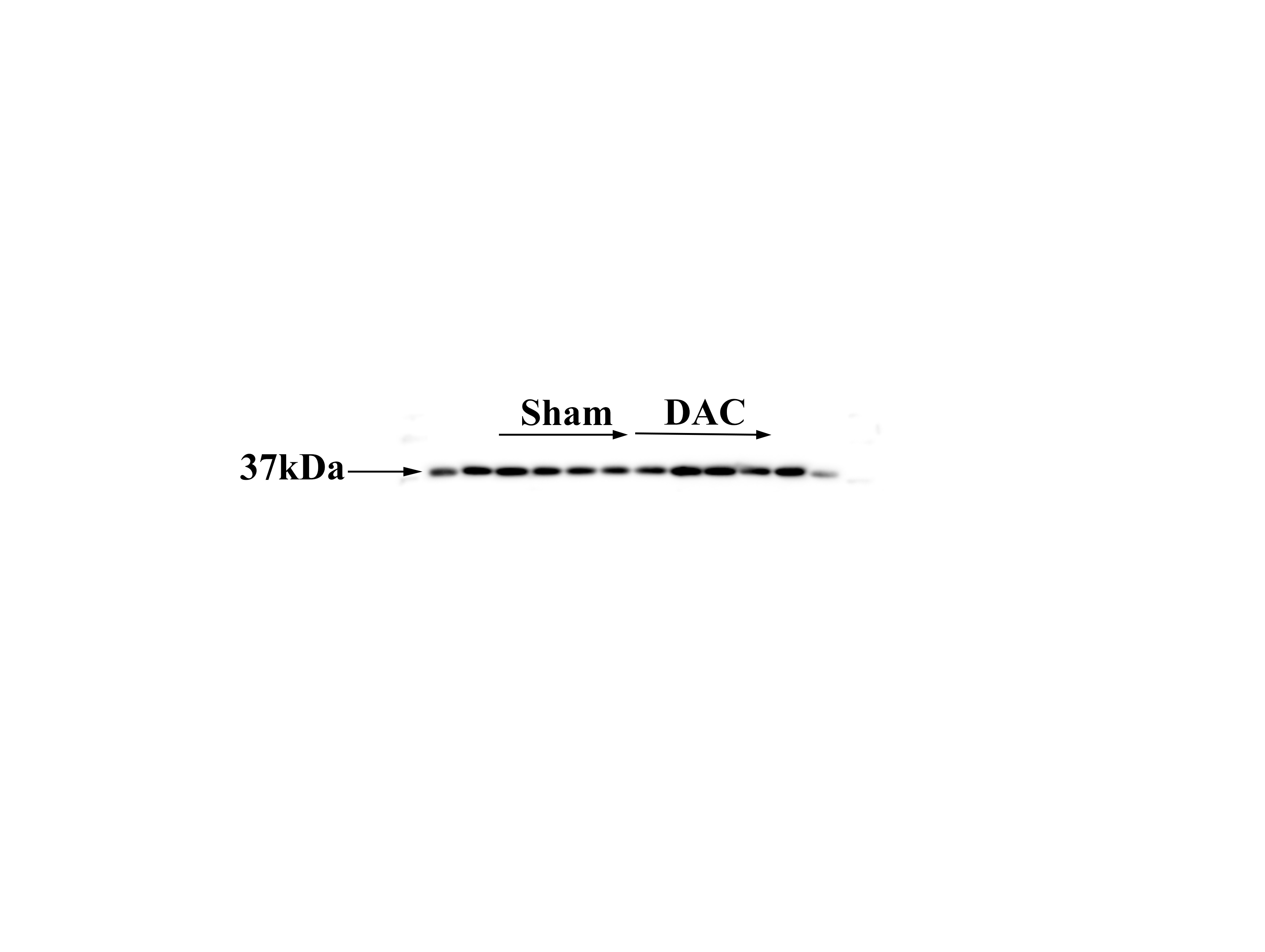

Supplement: Supplementary file 1 [file Data_Sheet_1.ZIP › Membranes for WB/9 GAPDH for p-PI3K.tif]

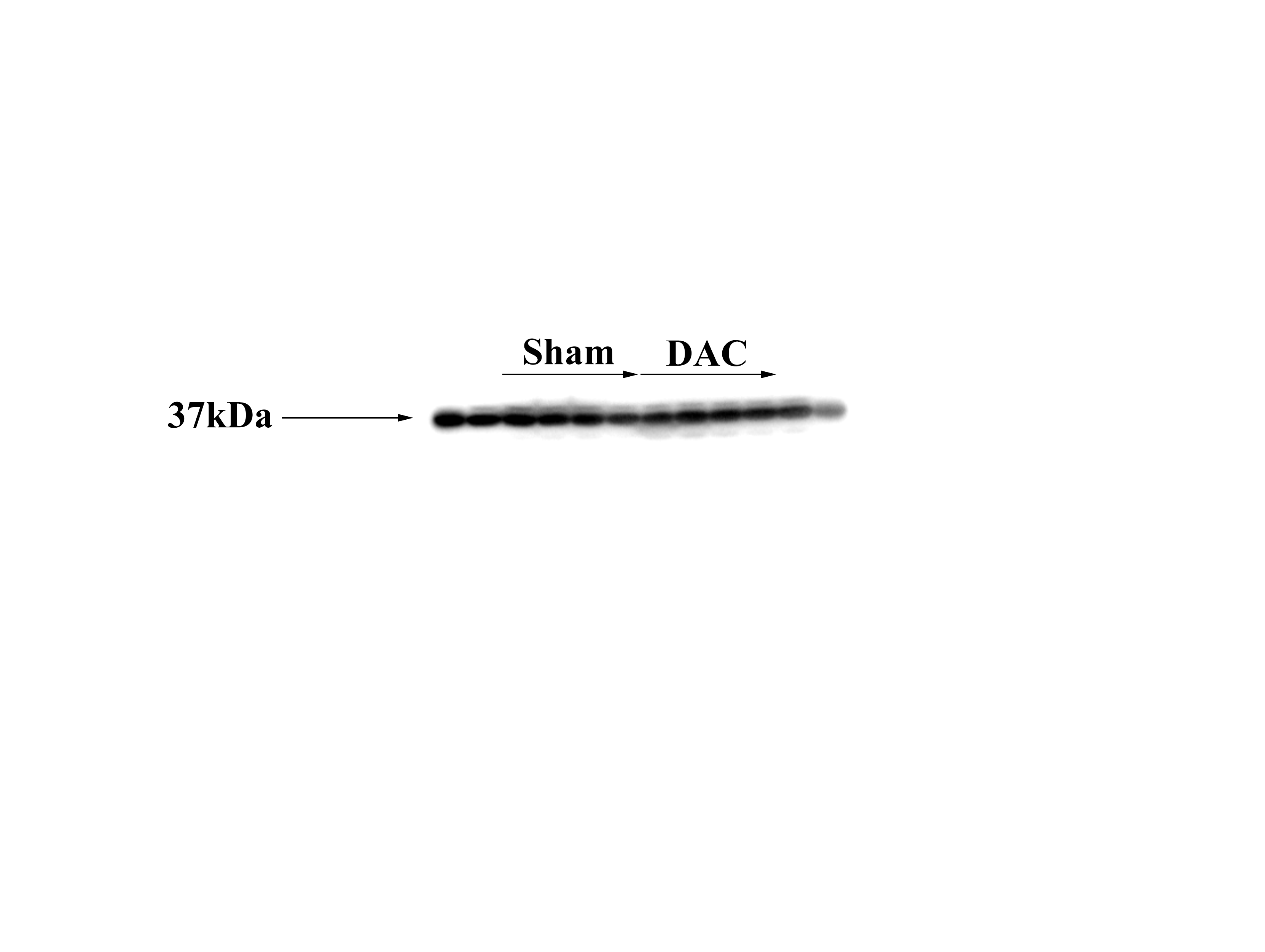

Supplement: Supplementary file 1 [file Data_Sheet_1.ZIP › Membranes for WB/7 GAPDH for p-Smad2.tif]

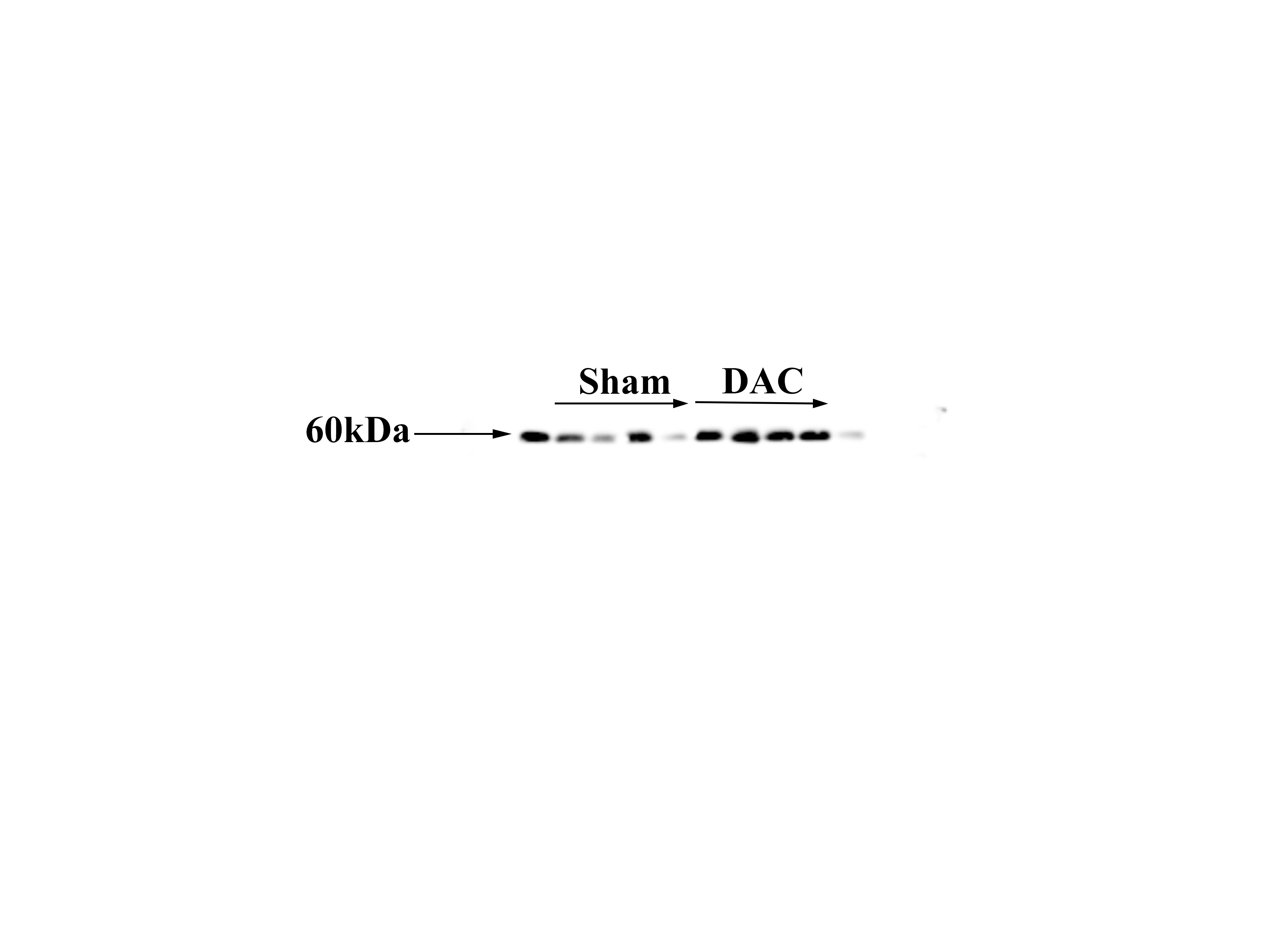

Supplement: Supplementary file 1 [file Data_Sheet_1.ZIP › Membranes for WB/8 p-Samd2-3.tif]

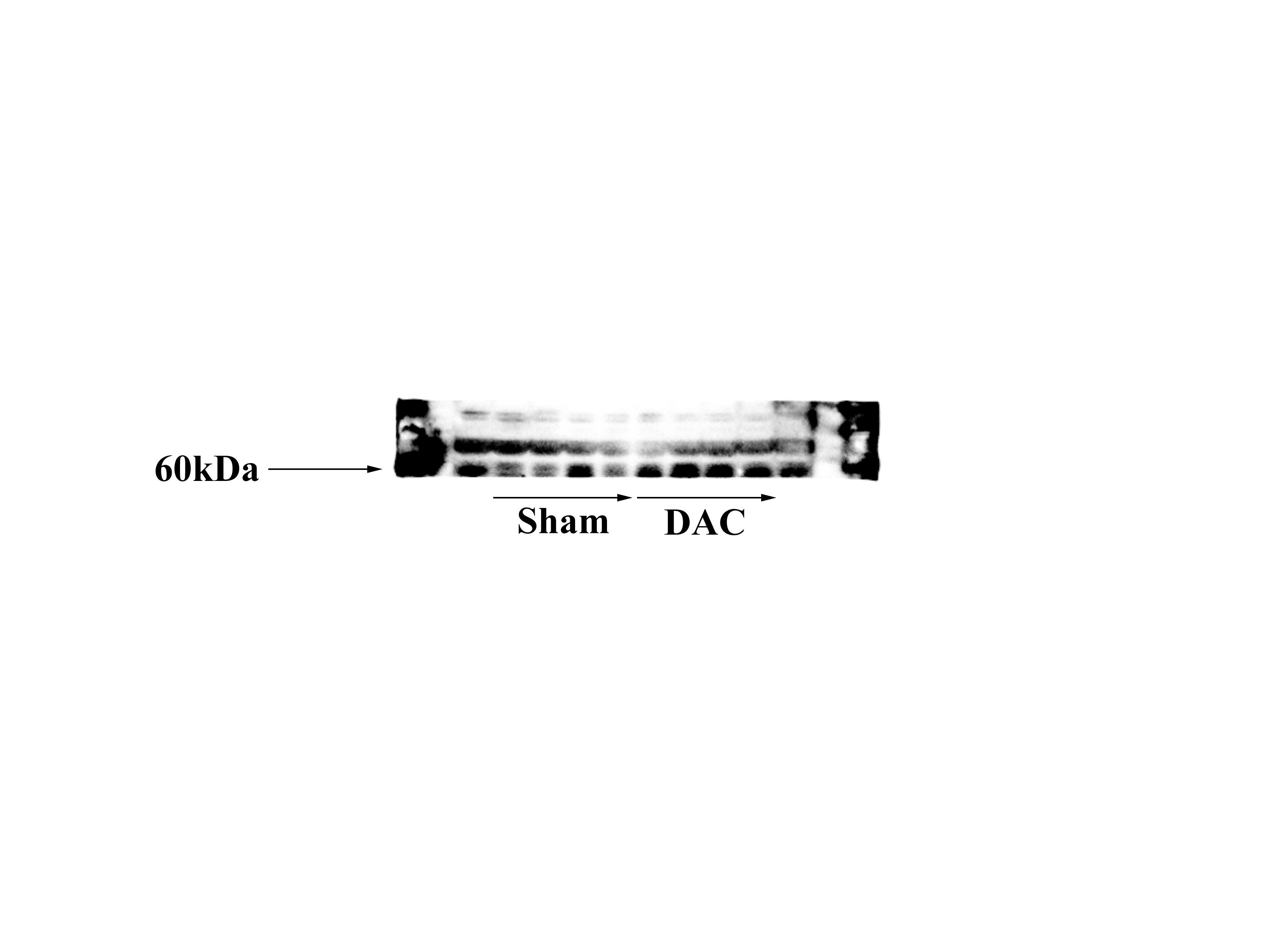

Supplement: Supplementary file 1 [file Data_Sheet_1.ZIP › Membranes for WB/7 p-Smad2.tif]

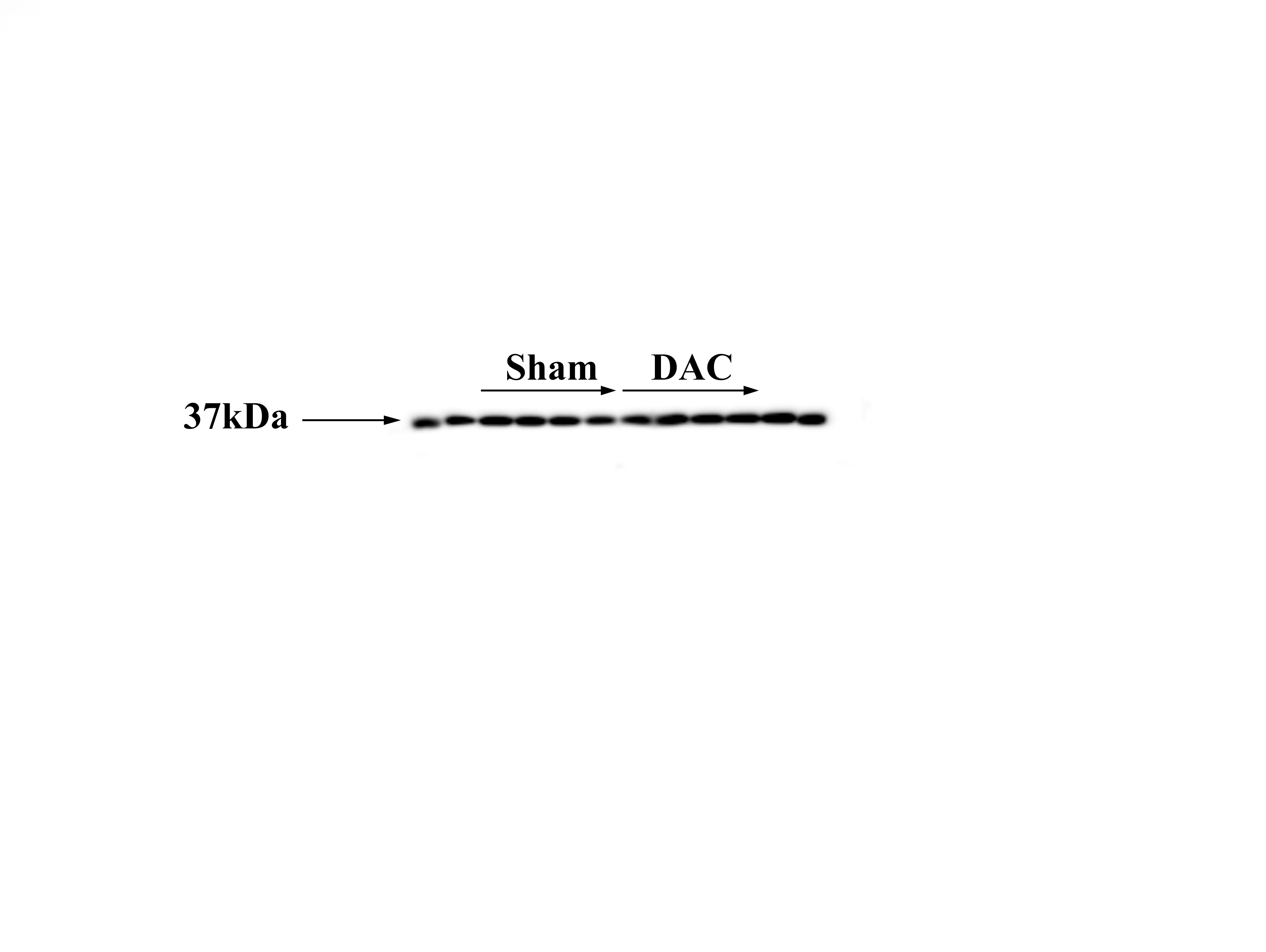

Supplement: Supplementary file 1 [file Data_Sheet_1.ZIP › Membranes for WB/8 GAPDH for p-Samd2-3.tif]

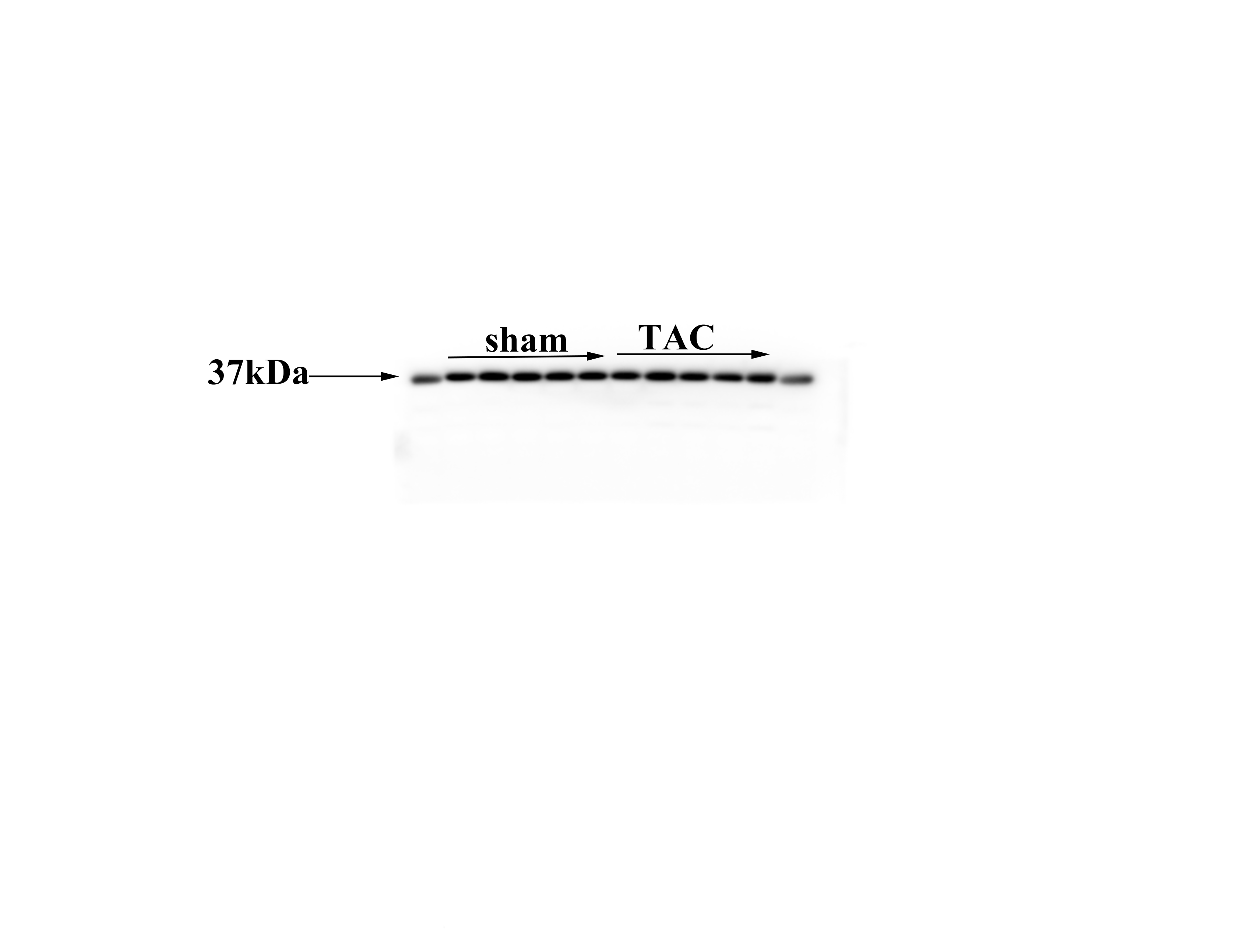

Supplement: Supplementary file 1 [file Data_Sheet_1.ZIP › Membranes for WB/5 gapdh for col1a┴1.tif]

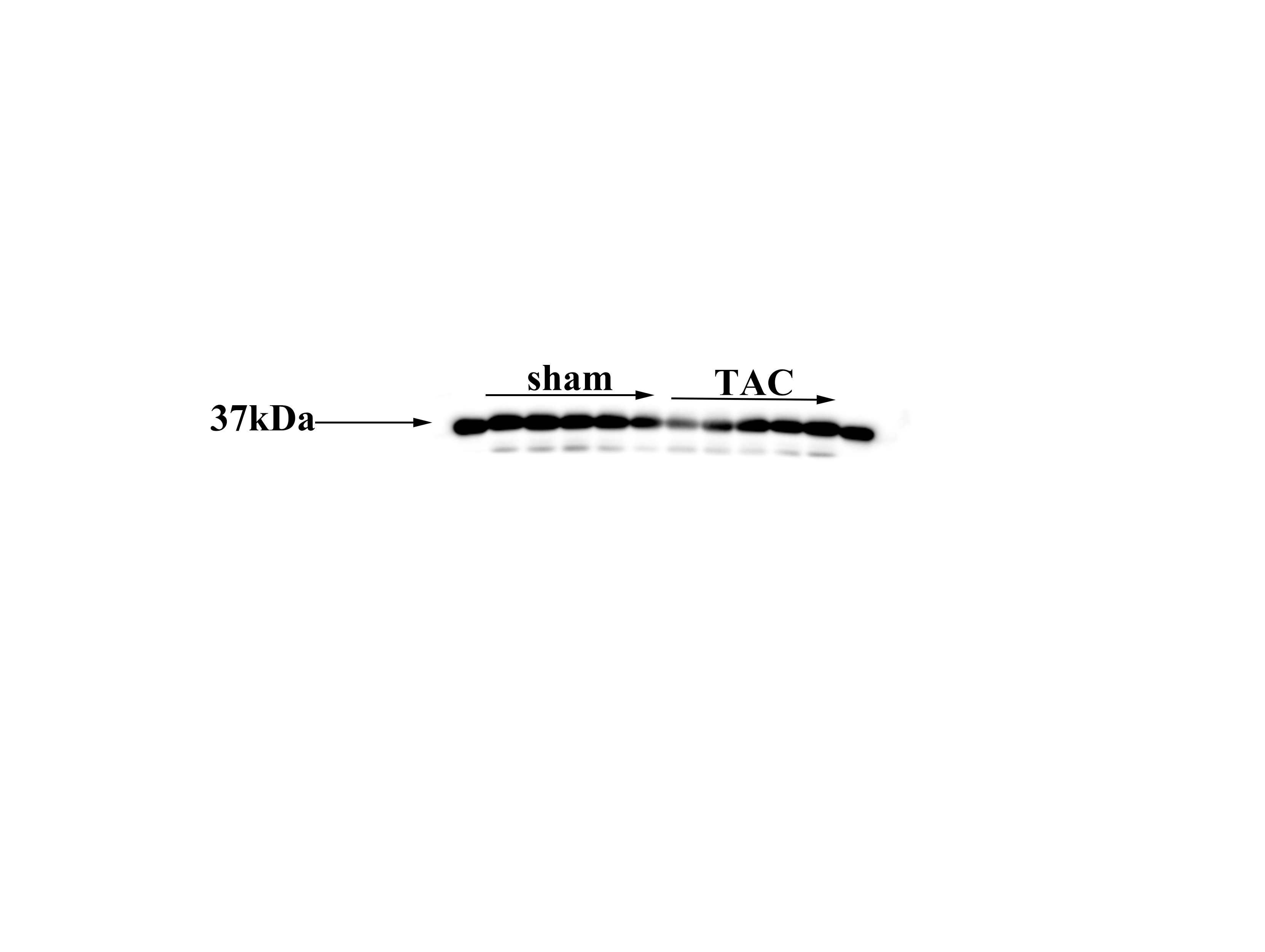

Supplement: Supplementary file 1 [file Data_Sheet_1.ZIP › Membranes for WB/7 GAPDH for p-Samd2.tif]

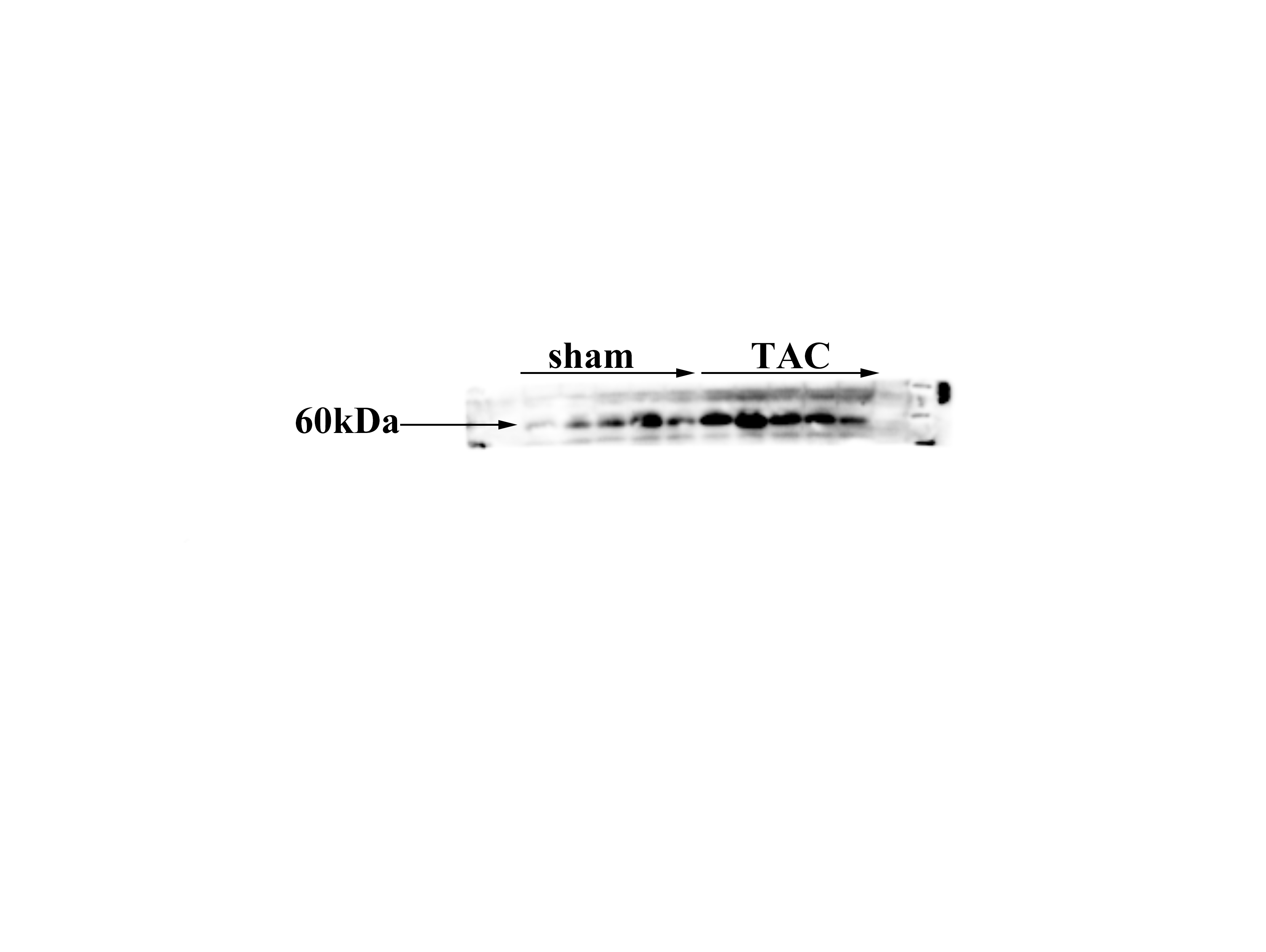

Supplement: Supplementary file 1 [file Data_Sheet_1.ZIP › Membranes for WB/7 p-Samd2.tif]
